# Assessing Gamma Index Sensitivity to Selected Linear Accelerator Mechanical Errors Using Various Dosimetric Systems

**DOI:** 10.3390/jcm15187183

**Published:** 2026-09-16

**Authors:** Hande Bas Ayata, Gokhan Aydin, Emrah Gokay Ozgur, Zeynep Ozen, Talip Celik, Ozcan Gundogdu

**Affiliations:** 1 Department of Biomedical Engineering, Institute of Natural and Applied Sciences, Kocaeli University, 41001 Kocaeli, Turkey; celiktlp@gmail.com (T.C.); o.gundo@gmail.com (O.G.); 2 Department of Radiation Oncology, Acıbadem Maslak Hospital, 34457 Istanbul, Turkey; gokhan.aydin@acibadem.com; 3 Department of Biostatistics, Marmara University Hospital, 34899 Istanbul, Turkey; emrahgokayozgur@gmail.com; 4 Department of Radiation Oncology, Acıbadem Antunizade Hospital, 34662 Istanbul, Turkey; zeynepozen68@yahoo.com

**Keywords:** gamma index, patient-specific quality assurance, VMAT

## Abstract

**Background:** Volumetric modulated arc therapy (VMAT) for stereotactic body radiotherapy (SBRT) involves highly modulated dose distributions and steep dose gradients, increasing the importance of patient-specific quality assurance (QA). Gamma index analysis is widely used for comparing measured and treatment planning system (TPS)-calculated dose distributions; however, its sensitivity to small systematic mechanical errors remains controversial, particularly across different detector systems and normalization methods. This study aimed to investigate the sensitivity of various gamma index criteria to intentional linear accelerator-based mechanical errors, including multileaf collimator (MLC) positional deviations and collimator rotation errors, using four dosimetric systems. Additionally, global and local gamma analyses were compared to evaluate their ability to detect clinically relevant geometric deviations in SBRT VMAT. **Methods:** Twenty-five lung SBRT VMAT plans were retrospectively selected. For each patient, six additional plans containing systematic MLC aperture widening errors (0.25, 0.5, 1, and 2 mm) and collimator rotation errors (0.5° and 2°) were generated, resulting in 175 treatment plans. Each plan was delivered to five different patient-specific QA dosimetric systems, resulting in a total of 875 QA measurements. Patient-specific QA measurements were performed using EPID-based EPIQA, ArcCHECK (SNCPATIENT/3DVH), COMPASS (Matrixx Evolution), and Octavius 4D systems. Gamma analyses were conducted using 3%/3 mm, 3%/2 mm, 2%/2 mm, 2%/1 mm, 1%/2 mm, and 1%/1 mm criteria with a 10% dose threshold. Pearson correlation analysis assessed relationships between global and local gamma results. Linear regression slopes quantified sensitivity to MLC aperture widening errors. Receiver operating characteristic (ROC) curves and area under the curve (AUC) values were calculated to evaluate error detectability. **Results:** Gamma sensitivity varied considerably depending on the characteristics of the dosimetric system (including detector resolution, reconstruction algorithm, and evaluation method) as well as the selected gamma criteria. The 2%/1 mm criterion consistently demonstrated high sensitivity across all systems, providing strong discrimination of ≥0.5 mm MLC errors and near-perfect AUC values for ≥1 mm deviations. The 1%/1 mm criterion yielded the steepest regression slopes but produced unstable passing rates, including in error-free plans, particularly in lower-resolution systems. Conventional 3%/3 mm and 3%/2 mm criteria frequently failed to detect clinically relevant submillimeter MLC deviations. EPID-based EPIQA showed the highest overall sensitivity and AUC performance for small errors, whereas ArcCHECK and Octavius 4D exhibited reduced responsiveness to subtle deviations. **Conclusions:** Gamma index performance in SBRT VMAT QA is strongly dependent on detector characteristics and gamma configuration. Among the evaluated criteria, 2%/1 mm provided the most balanced combination of sensitivity and clinical stability. Widely used 3%/3 mm thresholds were insufficient for detecting submillimeter geometric deviations. Detector-specific and technique-specific gamma protocols should be implemented to ensure reliable identification of clinically relevant mechanical errors in high-precision radiotherapy.

## 1. Introduction

Volumetric Modulated Arc Therapy (VMAT) [1] is a recent technique in which the gantry rotates while the beam aperture adjusts continuously; the dose rate and gantry speed are also variable. Due to the complexity of advanced delivery systems compared to conventional radiotherapy, specialized quality assurance (QA) programs are required [2,3,4,5,6,7,8,9]. In this sense, the patient QA procedure generally allows clinically significant errors that may occur at any point in the treatment chain to be detected. Therefore, patient-specific QA is necessary to verify the fluence delivered by the VMAT beams and ensure the MLC model’s accuracy for each VMAT plan before the treatment. Several QA devices are commercially available for enabling dose evaluation by comparing planned dose distribution to the measured dose delivered. However, the clinical tolerances, applicability, and limitations of each QA device can vary across different treatment modalities. This variability arises from the challenge of evaluating the contributions of different system components. One such difference is the structural characteristics of the detector and their impact on the measured signal or dose. Additionally, the data correction or processing methods applied to raw measurement signals can vary. Moreover, the dose calculation models used by the systems also differ [10,11,12]. Finally, there are dose comparison metrics, such as the widely used gamma index method implemented in clinical and commercial software. The application of gamma analysis can be significantly affected by detector resolution combined with other characteristics.

The commonly used method in VMAT plan quality control is gamma index analysis [13,14]. In clinical practice, such analysis is frequently used to assess the agreement between calculated and measured dose distributions. This method incorporates two key parameters: the percent dose difference (DD) and the distance to agreement (DTA). There are two primary approaches to calculating DD within the gamma index framework: global and local gamma index analyses [14]. In global gamma analysis, the dose difference is normalized to the maximum dose within the evaluation area. In local gamma analysis, the dose difference is normalized to the local planned dose. These different normalization approaches affect the final gamma passing rate by changing the sensitivity of the analysis in both low- and high-dose regions. In the absence of explicit guidelines for employing global or local gamma index analysis in pre-treatment QA for VMAT, individual clinics independently determine which gamma index analysis method to utilize. However, it remains uncertain whether global gamma index analysis can replace local gamma index analysis. Caution is therefore necessary when determining the gamma analysis parameters for plan quality control. Moreover, previous studies have identified certain challenges associated with the gamma index method. These challenges include the computational intensity of the gamma index calculation [7,9,11,12], which can be time-consuming, and the impact of factors such as interpolation, search distance, and resolution on analysis accuracy. Additionally, the effectiveness of the gamma index analysis as a radiotherapy verification tool in radiotherapy has been questioned, with concerns about its reliability in detecting clinically significant dose discrepancies [8]. These issues highlight the need for careful consideration and potential refinement to ensure the method’s efficacy in clinical settings. It is therefore necessary to be careful when determining the gamma analysis parameters for plan quality control.

Several studies have investigated the sensitivity of gamma index analysis to potential delivery errors, including MLC, couch, gantry, and collimator position errors. The aim of these studies is to reveal the capabilities of dosimetric equipment and gamma analysis techniques. Kim et al. examined the changes in gamma passing rates in response to three types of MLC errors and found that the global gamma criterion at 2%/1 mm was more sensitive in detecting errors [15]. Heilmann et al. compared the 2%/2 mm gamma passing rate with the 3%/3 mm passing rate in 2D gamma evaluation for SBRT (Stereotactic Body Radiation Therapy) treatments in VMAT QA, demonstrating that the 2%/2 mm gamma criterion showed higher sensitivity in detecting errors for pre-treatment QA [3]. This work concentrates on use of four measurement devices: ArcCHECK, as a three-dimensional diode cylinder (Sun Nuclear); PTW 2D ARRAY 729, as a two-dimensional ion chamber array combined with the Octavius 4D; COMPASS with MatriXX detector array (IBA Dosimetry, Schwarzenbruck, Germany); and EPIQA, an electronic portal imaging device (EPID) associated with EPIQA (EPIDos).

In this study, we investigated the sensitivity of the gamma index method with various gamma criteria by introducing intentional MLC errors. The local gamma index tends to reveal errors more clearly in high-dose gradient and low-dose regions, whereas the global gamma index may mask such errors while emphasizing agreement in higher-dose regions within the dose distribution [8,16]. The aim of this study is therefore to also evaluate the ability of each of the four devices used to detect intentional errors by comparing the results of their global and local gamma analyses. There are a limited number of studies that evaluate the sensitivity to MLC and collimator rotation errors using six different gamma index analyses, both globally and locally, across four different dosimetry systems. In this study, gamma analysis-both global and local-was conducted across all dosimetric systems except for the COMPASS, using six distinct gamma criteria (3%/3 mm, 3%/2 mm, 2%/2 mm, 2%/1 mm, 1%/2 mm and 1%/1 mm) with a 10% dose threshold. The results were comparatively evaluated to assess performance under varying levels of stringency. Additionally, for each detector system, area under the curve (AUC) values corresponding to different gamma criteria were calculated by considering these systematic errors. The obtained AUC values were statistically analyzed, and the detection sensitivity of different error types across detector systems was compared. Within the scope of these analyses, Receiver Operating Characteristic (ROC) curves were generated, and the performance of the detectors under various error scenarios was examined both visually and quantitatively.

## 2. Materials and Methods

### 2.1. Generation of VMAT Plans for Stereotactic Body Radiotherapy

A total of 25 patients with lung cancer treated with the Stereotactic Body Radiotherapy (SBRT) technique in 5 fractions were retrospectively selected for this study. All reference treatment plans were calculated in the Eclipse Treatment Planning System (Version 13.6; Varian Medical Systems, Palo Alto, CA, USA) using the Analytical Anisotropic Algorithm (AAA), with tissue heterogeneity correction enabled based on the patient CT datasets with a grid size of 1 × 1 × 1 mm^3^. EPIQA performed detector-level two-dimensional dose verification and did not reconstruct the dose distribution within the patient anatomy. Treatment plans were designed using either 2 full arcs or 2 half arcs, depending on tumor location, with 6 FFF photon beams at a dose rate of 1400 MU/min. Prescription doses to target volumes (PTVs), including the primary tumor, were 60 Gy in 5 fractions. The Minimum Leaf Gap parameter configured in the Eclipse TPS was 0.5 mm.

Patient-specific QA measurements were performed using the clinical treatment plan for a single treatment fraction. Before measurements, all QA systems underwent routine calibration and performance verification, including detector response checks, in accordance with the manufacturers’ recommendations and the institutional QA protocol. All detector array systems were calibrated on the same linear accelerator used for the subsequent patient-specific QA measurements, thereby inherently compensating for any potential drift in linac output. In addition, daily machine quality assurance, including beam output constancy verification, was performed before all patient-specific QA measurements. Beam output constancy was required to remain within the institutional action level of ±2% from the baseline value before patient-specific QA measurements were initiated. ArcCHECK 3DVH and COMPASS reconstructed the dose distribution using their respective reconstruction algorithms, whereas the Octavius 4D system was used without the DVH Anatomy module; therefore, dose reconstruction was performed within the Octavius phantom rather than the patient anatomy.

The jaw tracking option was disabled for both the error-free reference plans and all intentional MLC aperture widening plans to ensure a consistent comparison and to isolate the dosimetric effect of the introduced MLC aperture widening errors.

### 2.2. Simulation of MLC Errors in VMAT Plans for SBRT

**Figure 1:**
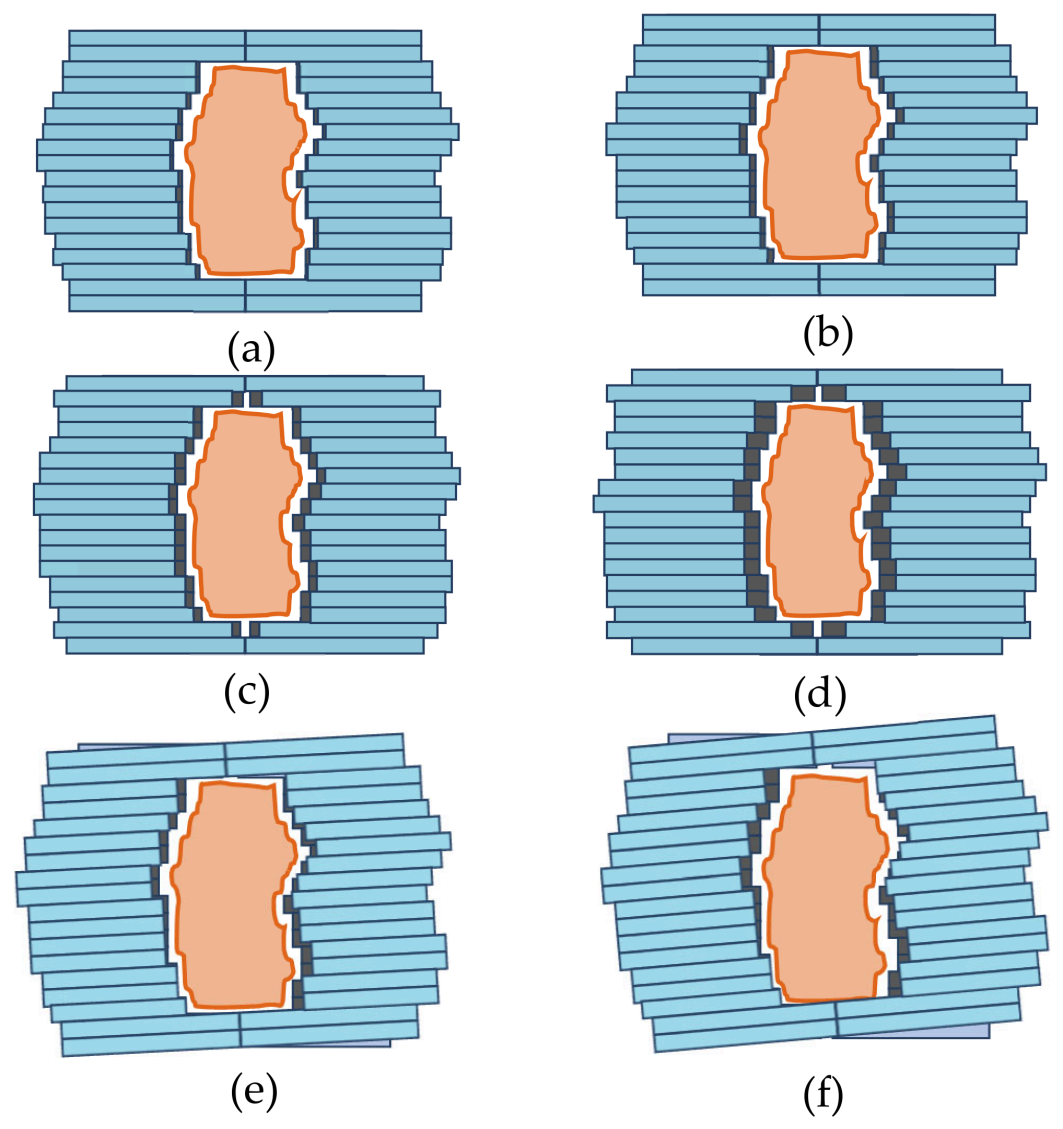
Representative illustration of MLC and collimator error patterns: (**a**) 0.25 mm outward systematic displacement applied to each MLC bank, (**b**) 0.5 mm outward systematic displacement applied to each MLC bank, (**c**) 1 mm outward systematic displacement applied to each MLC bank, (**d**) 2 mm outward systematic displacement applied to each MLC bank, (**e**) 0.5° collimator rotation error, and (**f**) 2° collimator rotation error.

All VMAT plans were transferred from the treatment planning system in DICOM format to a homemade software developed in C# 4.5 (Visual Studio 2013), which allowed modifications of the MLC aperture widths through symmetric outward shifts in both MLC leaf banks, as well as collimator rotations. Each arc in the VMAT plans consisted of 177 control points. Each of these contained information about the positions of all MLC leaves; beam apertures thus changed shape independently from one control point to the next. MLC errors were systematically simulated in all control points of each field using the software, and the MLC openings were systematically distorted for each opposing MLC bank. Each opposing MLC bank was displaced outward by 0.25 mm, 0.50 mm, 1 mm, and 2 mm relative to the original treatment plan; the stated values therefore denote the per-bank leaf displacement, and the resulting total aperture change is twice these values (Figure 1). The applied MLC modification represents a systematic calibration (gap-opening) error in which opposing leaves from both MLC banks were displaced symmetrically by the same magnitude. This error model was deliberately selected because it provides a reproducible and controlled perturbation with a monotonic dosimetric response, allowing consistent evaluation of gamma index sensitivity across different dosimetric systems. Asymmetric single-bank, individual leaf, or stochastic leaf-position errors were beyond the scope of this study. This configuration represents a systematic calibration- or gap-type MLC error rather than asymmetric single-bank, individual-leaf, or random per-leaf positioning errors; systematic errors were selected deliberately because their reproducible, monotonic dose–response permits quantitative characterization of detector sensitivity through regression slope and ROC/AUC analysis. Asymmetric single-bank, individual leaf or stochastic leaf-position errors were beyond the scope of the present study.

**Figure 2:**
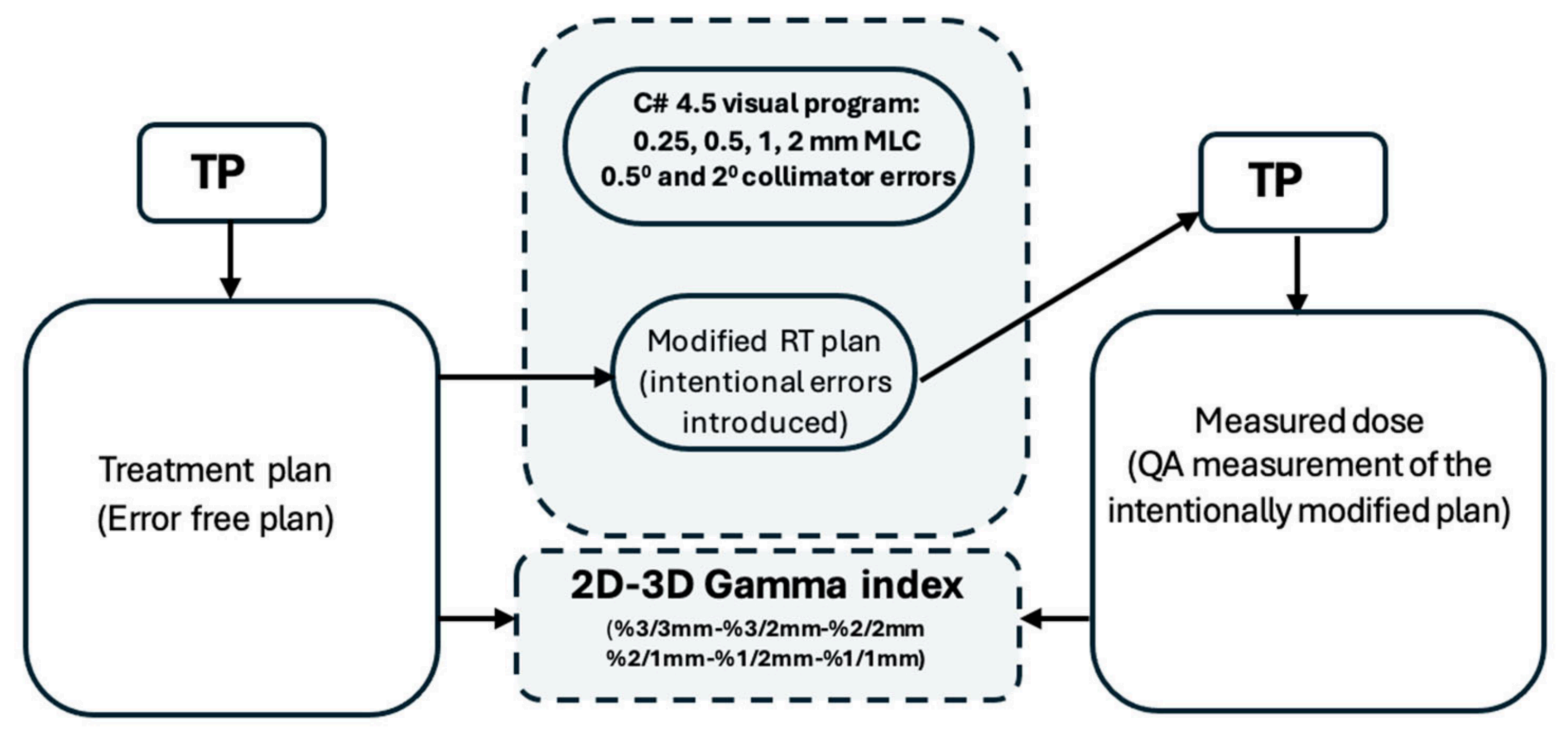
Schematic representation of the error evaluation workflow.

Additionally, collimator rotation errors of 0.5° and 2° were introduced at each control point. The 0.5° rotation error was included to evaluate the sensitivity of the dosimetric systems to small rotational deviations that may occur in clinical practice and may be challenging to detect using conventional patient-specific QA. In total, seven plans were generated for each patient, including the original error-free treatment plan, resulting in a total of 175 plans. The several gamma evaluation results of the original treatment plans QAs were compared with the plans with intentional errors for each patient. A schematic representation of the study’s error evaluation workflow is shown in Figure 2.

### 2.3. QA Systems

In this study, four types of dosimetry systems, EPID-based EPIQA (EPIdos S.r.l., Castel Gandolfo, Rome, Italy), ArcCHECK™ (Sun Nuclear Corporation, Melbourne, FL, USA), COMPASS (IBA Dosimetry GmbH, Schwarzenbruck, Germany), and PTW Octavius 4D (PTW-Freiburg GmbH, Freiburg, Germany), were evaluated, namely their differing sensitivities to systematic intentional errors introduced in each VMAT plan. CT scans of each dosimetry system were acquired to generate 3D dose distributions, and these scans were used to create verification plans in the Eclipse treatment planning system. When calculating the reference 2D and 3D dose distributions in the Eclipse system for gamma evaluation, the calculation grid was 1 mm. Before pre-treatment QA, the absolute dose of arrays and their relative reading were calibrated for each system.

### 2.4. EPID-Based EPIQA

In EPID-based EPIQA, the EPID is used to perform quality assurance by capturing images that reflect the actual radiation dose delivered to the patient. The acquired images are analyzed by specialized software that compares the measured dose distribution (from EPID images) to the planned dose distribution generated by the TPS. The software calculates any deviations or discrepancies between the expected and actual dose distributions. After the comparison, the results are typically visualized in the form of dose maps or gamma analysis, which quantify the agreement between the planned and delivered doses [17].

### 2.5. ArcCHECK

The ArcCHECK system consists of 1386 n-Si diodes arranged on a cylindrical surface with a diameter of 21 cm, housed within a ring-shaped PMMA phantom. The detectors are spaced 1 cm apart, forming 21 helical rings, each containing 66 detectors. The 3D dose matrix calculated by the treatment planning system is imported into SNC software and unfolded into a 2D dose plane covering all detectors. The ArcCHECK 3DVH uses the standard ArcCHECK detector array, but it works in conjunction with 3DVH software to analyze dose measurement data. The software processes the measured 2D dose data and compares it to the planned dose distribution to generate a 3D dose distribution estimation. For each verification plan, a single ArcCHECK measurement was acquired and subsequently analyzed using both SNC Patient and 3DVH software. Therefore, no additional treatment delivery was required [18].

### 2.6. COMPASS

The IBA Compass system works with a device known as Matrixx Evolution, which consists of a matrix of detectors positioned to measure the radiation dose delivered during treatment. These detectors are typically embedded within a phantom or placed in direct alignment with the patient to obtain accurate readings. After collecting the data, the system uses advanced software tools to analyze and evaluate the dose distribution. The software called COMPASS (Version 4.1) provides detailed analysis tools, including gamma analysis and dose distribution comparisons, ensuring thorough evaluation of the delivered dose. The Compass software receives this data from the Matrixx Evolution and compares it with the planned dose distribution from the TPS. This comparison allows for the detection of discrepancies between the expected and actual dose distribution. The software typically uses methods such as gamma analysis to quantify the agreement [19]. During the study, local gamma analysis could not be performed with the COMPASS system despite repeated attempts. The issue was reported to the manufacturer; however, the exact technical cause could not be definitively identified within the study period. Therefore, only global gamma analysis results were included in this study.

### 2.7. Octavius 4D

The Octavius 4D system (PTW-Freiburg GmbH, Freiburg, Germany) includes a two-dimensional array of 729 ionization chambers embedded within a rotational phantom for patient-specific quality assurance. The measured dose data are analyzed using VeriSoft software, which reconstructs the three-dimensional dose distribution within the Octavius phantom and compares it with the TPS-calculated dose distribution using gamma analysis and dose distribution comparison. The Octavius 4D system was used without the DVH Anatomy module; therefore, dose reconstruction was performed within the phantom rather than in the patient anatomy [20].

### 2.8. Data Analysis

Statistical analysis was performed using the IBM SPSS 20.0 software package (IBM Corp., Armonk, NY, USA). Numerical variables that followed a normal distribution were presented as mean ± standard deviation, while categorical variables were expressed as frequency (percentage). To evaluate the consistency between global and local gamma passing rates, the Pearson correlation coefficient (r) was calculated with corresponding *p*-values for various gamma criteria. Correlation values (r) between 0.3 and 0.49 were considered weak, while values from 0.5 to 0.69 and from 0.7 to 1.0 were regarded as moderate and strong correlations, respectively. In this study, *p*-values equal to or less than 0.05 were considered statistically significant. The correlations between all dosimetry systems used in the study were analyzed to evaluate gamma passing rates’ dependency on the dosimetry system. To assess the detectability of mechanical errors during VMAT delivery, correlations between gamma passing rates and original VMAT plans, and intentionally error plans were analyzed. Doses below 10% of the maximum dose were ignored to reduce the effect of statistically unstable low-dose voxels, in accordance with the AAPM TG-218 recommendation [18]. A uniform low-dose threshold was applied across all platforms so that observed inter-system differences would reflect intrinsic detector characteristics rather than differing low-dose noise behavior.

Gamma analyses were performed using the 3%/3 mm, 3%/2 mm, 2%/2 mm, 2%/1 mm, 1%/2 mm, and 1%/1 mm criteria with a 10% dose threshold. The selected gamma criteria included the clinically recommended 3%/2 mm criterion proposed in AAPM TG-218 together with stricter and more permissive criteria to systematically evaluate the influence of gamma criterion stringency on the detectability of small systematic MLC aperture widening errors in SBRT VMAT plans. For each dosimetric system, gamma analysis was performed using the manufacturer’s recommended clinical implementation and default software settings. No user-defined modifications were applied to the interpolation or search parameters. To quantitatively evaluate the relationship between MLC positional errors and gamma passing rates, a linear regression analysis was performed. For each verification system, the gamma passing rate was plotted as a function of the magnitude of the MLC deviation, and a best-fitting line was obtained according to the following model:

Gamma passing rate (%) = a + b × leaf (bank) positional error (mm).(1)

In this Equation (1), *a* represents the intercept, while *b* denotes the slope (or gradient) of the regression line, expressed in % per mm. The slope (*b*) characterizes the rate of change in gamma passing rate for each millimeter increase in leaf (bank) displacement. A more negative slope indicates a faster decline in gamma passing rate with increasing MLC error, thereby reflecting higher detector sensitivity to leaf positional deviations. Consequently, the slopes of the best-fitting lines serve as quantitative indicators for comparing the error detection capability of different dosimetric systems.

To compare the sensitivity and specificity of different gamma criteria across the evaluated dosimetric systems and intentional error magnitudes, ROC curves and AUC values were used. For ROC analysis, the error-free original plans were defined as the negative (reference) class, whereas plans containing intentional MLC or collimator errors were defined as the positive class. ROC analyses were performed separately for each dosimetric system, gamma criteria, and error magnitude. To avoid recommending a gamma index metric that is not statistically reliable, the area under the AUC was calculated for each metric. AUC values were calculated together with their 95% confidence intervals using IBM SPSS Statistics. The following guide was used to interpret the results: 1 = perfect test, 0.9 < AUC < 1 = highly accurate, 0.7 < AUC ≤ 0.9 = moderately accurate, 0.5 < AUC ≤ 0.7 = less accurate, and AUC = 0.5 = non-informative.

This retrospective study was approved by the Kocaeli University Non-Interventional Clinical Research Ethics Committee (KU GOKAEK; approval code: 2018/1613; approval date: 17 October 2018). All patient data used in the study were anonymized prior to analysis.

## 3. Results

**Figure 3:**
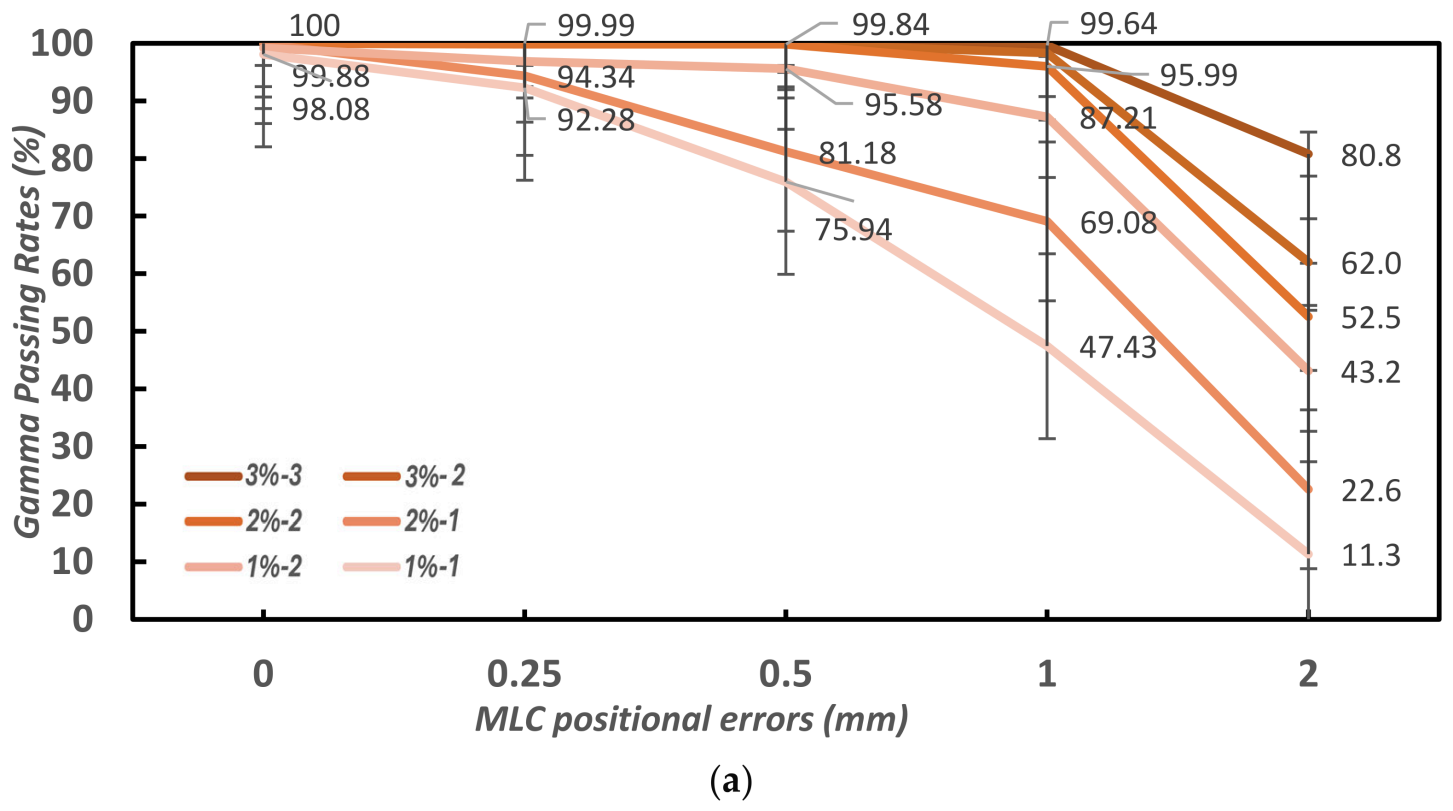

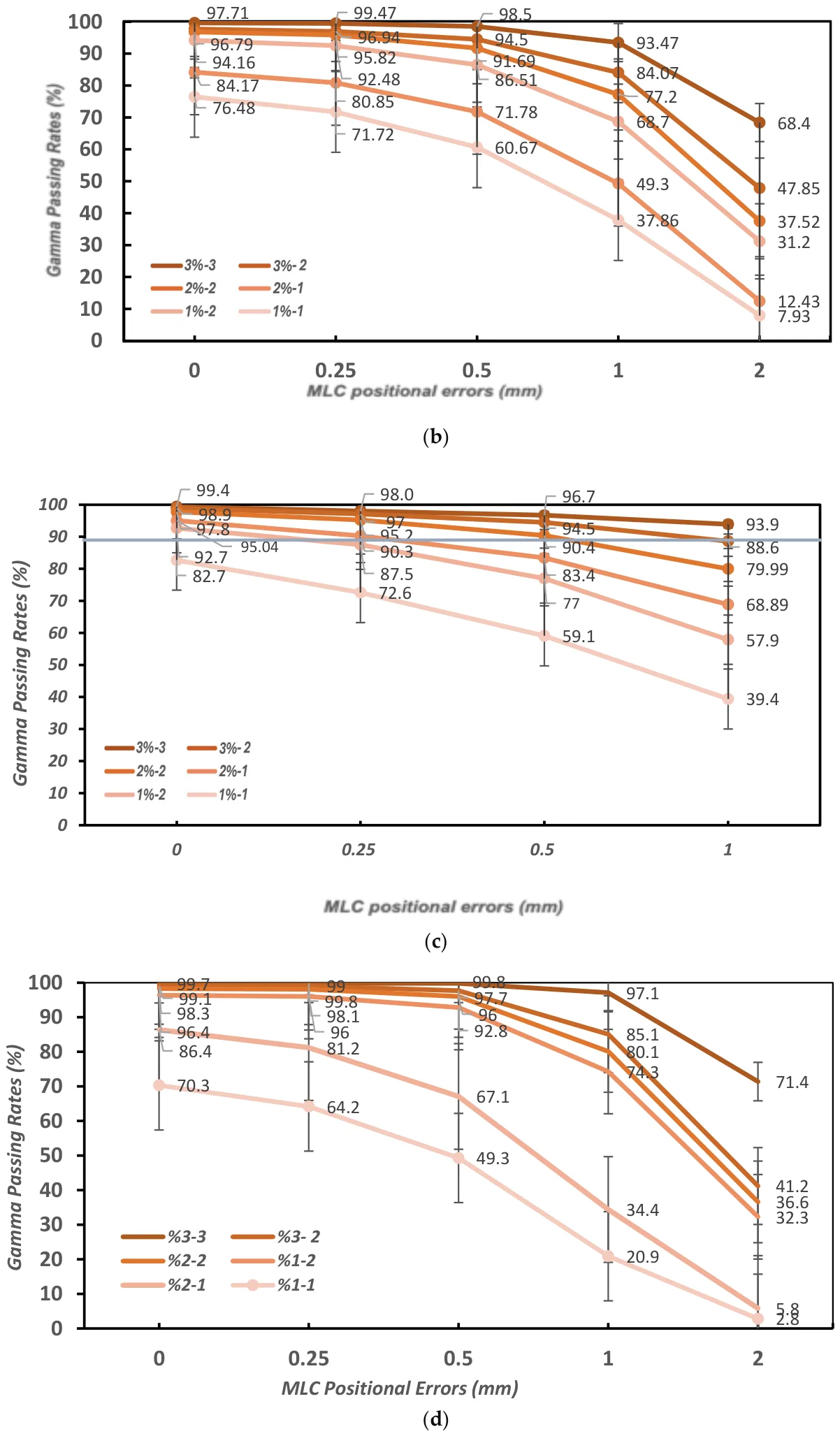

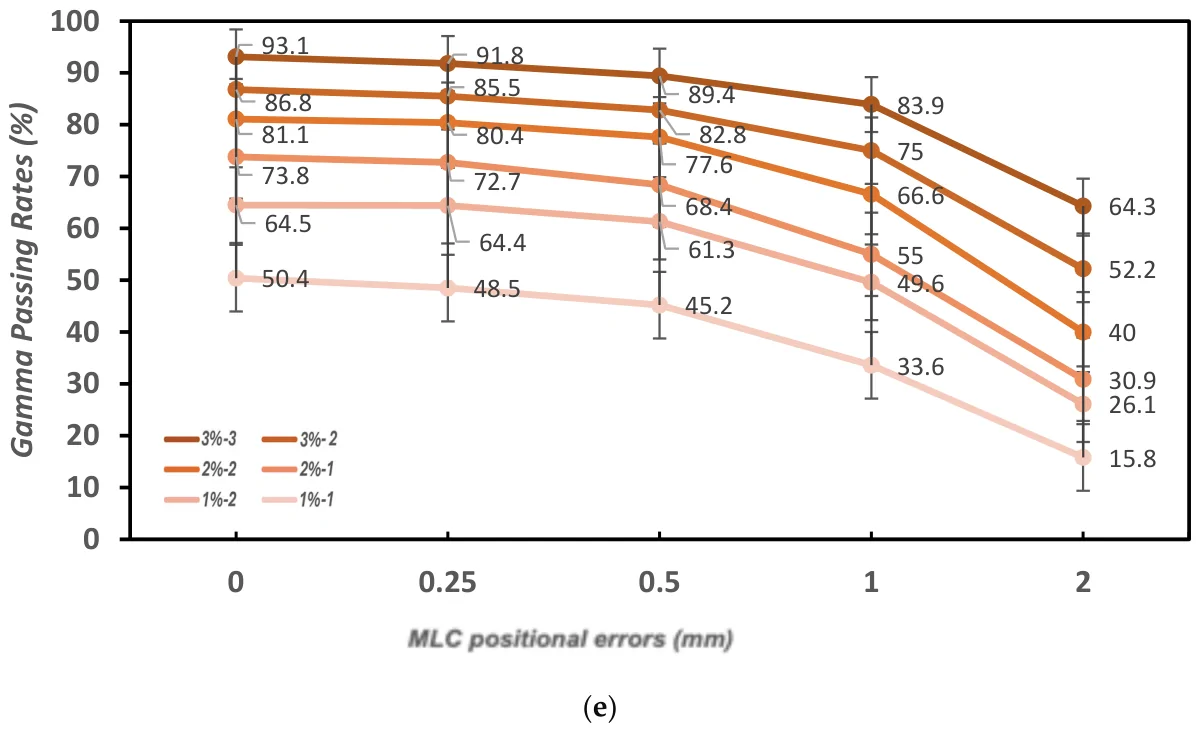
(**a**) Gamma passing rates against MLC aperture widening errors in the EPIQA system. The x-axis is presented on a logarithmic scale. Error bars represent the standard deviation of the gamma passing rates. (**b**) Gamma passing rates against MLC aperture widening errors in the SNCpatient system. The x-axis is presented on a logarithmic scale. Error bars represent the standard deviation of the gamma passing rates. (**c**) Gamma passing rates against MLC aperture widening errors in the ArcCHECK-3DVH system. The x-axis is presented on a logarithmic scale. Error bars represent the standard deviation of the gamma passing rates. (**d**) Gamma passing rates against MLC aperture widening errors in the COMPASS system. The x-axis is presented on a logarithmic scale. Error bars represent the standard deviation of the gamma passing rates. (**e**) Gamma passing rates against MLC aperture widening errors in the Octavius 4D system. The x-axis is presented on a logarithmic scale. Error bars represent the standard deviation of the gamma passing rates.

**Figure 4:**
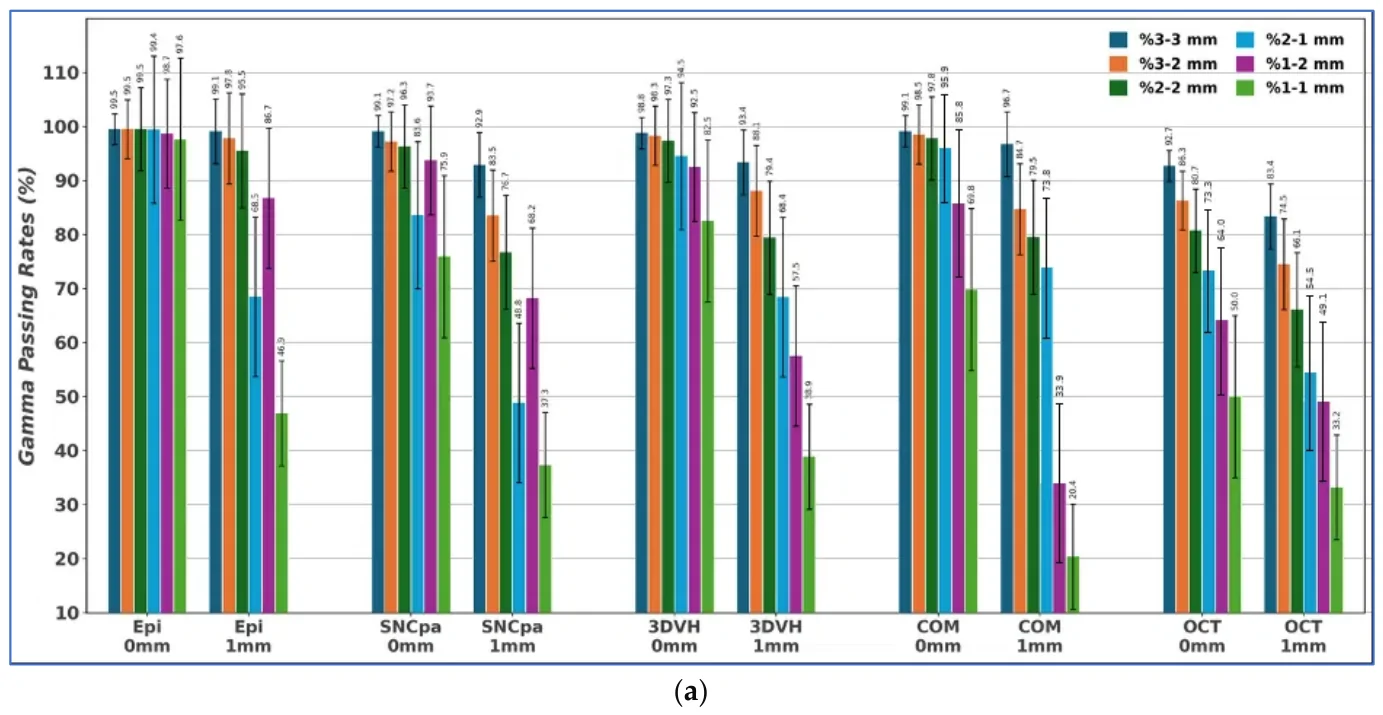

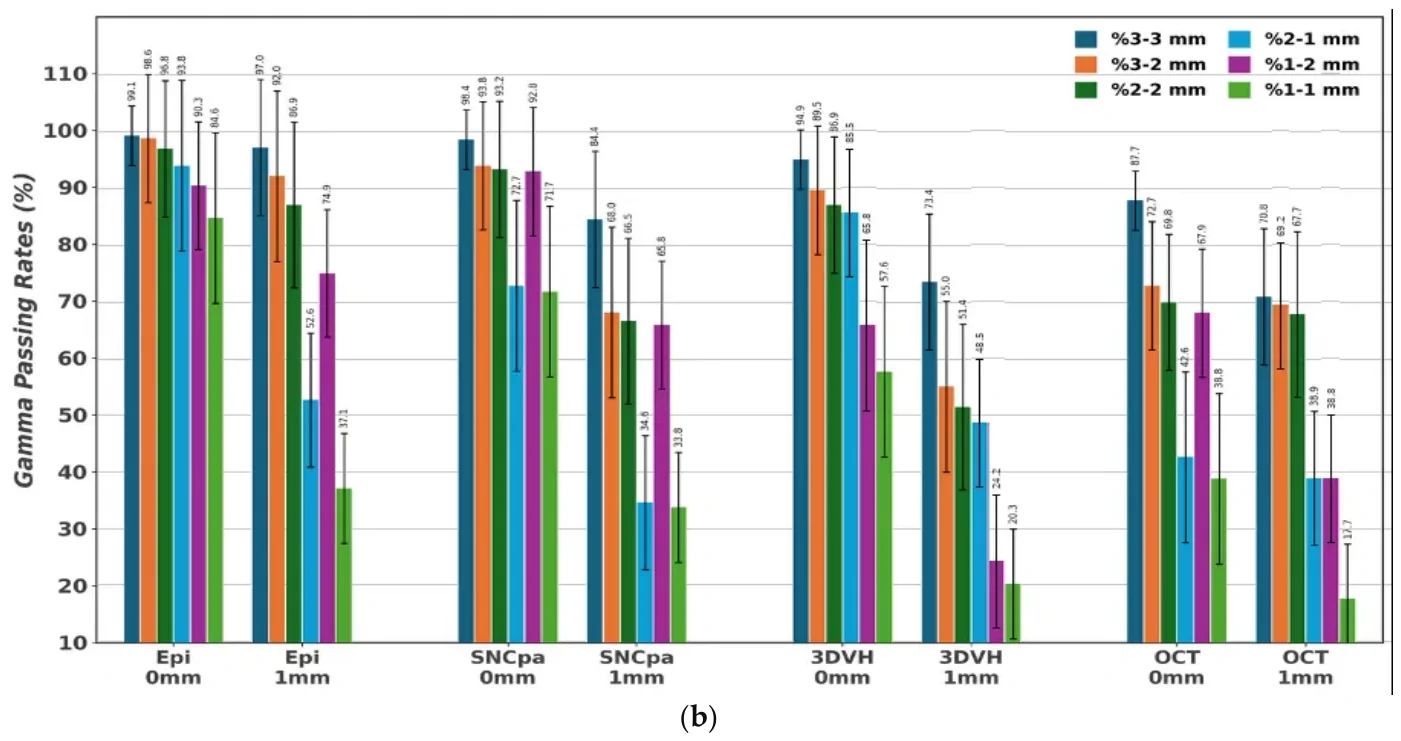
(**a**) Global gamma passing rates for different dosimetry system for error-free (MLC = 0 mm) and intentional error (MLC = 1 mm) SBRT VMAT treatment plans under different gamma criteria. Epi: EPID-based EPIQA, SNCpa: SNCPATIENT, 3DVH: ArcCHECK-3DVH, COM: COMPASS, OCT: Octavius dosimetry system. (**b**) Local gamma passing rates for different dosimetry system for error-free (MLC = 0 mm) and intentional error (MLC = 1 mm) SBRT VMAT treatment plans under different gamma criteria. Epi: EPID-based EPIQA, SNCpa: SNCPATIENT, 3DVH:3DVH, OCT: Octavius dosimetric system.

The relationship between different MLC aperture-widening errors and global gamma passing rates under different gamma criteria across the various dosimetric systems is shown in Figure 3a–e. Except for Figure 4b, all gamma passing rates plotted in the figures and listed in the tables were obtained using global gamma analysis. Figure 4b presents local gamma passing rates. For EPIQA, the gamma passing rates of the error-free plans were greater than 95% for all gamma criteria. When an 80% tolerance threshold was applied to the 1%/1 mm criterion, MLC aperture-widening errors of 0.5, 1, and 2 mm were detectable. The 2%/1 mm gamma criterion were found to be the second most sensitive gamma criteria for EPIQA, and errors after 0.5 mm were detectable for the 90% pass rate. The 3%/3 mm criterion had the lowest sensitivity, and the 3%/3 mm, 3%/2 mm, and 2%/2 mm gamma criteria were found to be unable to detect errors of 1 mm or smaller for the 90% passing rate. The 1%/2 mm gamma criterion were found to be unable to detect errors up to 0.5 mm for the 90% pass rate (Figure 3a).

The pass rates for SNCPATIENT were found to be lower than the EPIQA results. The pass rates of the original plan were found to be greater than 90% for the 3%/3 mm, 3%/2 mm, 2%/2 mm, and 1%/2 mm gamma criteria. For SNCPATIENT, the 2%/1 mm and 1%/1 mm criteria were the most sensitive and could detect MLC errors of 0.5 mm or larger at an 80% pass rate. For the 80% pass rate, the 1%/2 mm and 2%/2 mm gamma criteria were found to be able to detect errors after a 1 mm MLC misalignment (Figure 3b).

In the 3DVH results, the gamma pass rates of the original plan were found to be greater than 90% for all criteria except for the 1%/1 mm gamma criterion. If the tolerance level is accepted as 80% for the 1%/1 mm gamma criterion, errors larger than 0.25 mm may be identified. However, the original error-free plan may also fall below this threshold, indicating reduced specificity and the potential for false-positive classification. The 2%/1 mm criteria were also found to be the second most sensitive gamma criteria in the 3DVH results, and MLC misalignments of 0.25 mm or larger may be identified at the 90% pass rate. The 3%/3 mm and 3%/2 mm gamma criteria were found to be unsuccessful in detecting errors up to 1 mm for the 90% pass rate. The 1%/2 mm gamma criterion was also found to be unable to detect errors up to 0.5 mm (Figure 3c). For the 3DVH system, the 2-mm MLC aperture widening error was excluded from ROC analysis because the extremely low gamma passing rates under the strictest gamma criteria resulted in insufficient variability for reliable AUC calculation.

In the COMPASS results, the gamma pass rates of the original plan were found to be greater than 90% for all criteria except for the 1%/1 mm and 1%/2 mm gamma criteria. If a 90% passing rate is adopted as the tolerance level for the 1%/1 mm and 1%/2 mm criteria, errors larger than 0.25 mm may be distinguishable; however, the original error-free plan may also fall below this threshold, indicating reduced specificity and a potential for false-positive classification. The 2%/1 mm criterion was also found to be the second most sensitive gamma criteria in the COMPASS results, and MLC misalignments of 0.25 mm or larger can be detected for the 90% pass rate. The 3%/3 mm criterion was found to be unable to detect errors up to 2 mm, and the 3%/2 mm, 2%/2 mm, and 1%/2 mm gamma criteria were found to be unsuccessful in detecting errors up to 1 mm for the 90% pass rate. At the 80% tolerance level, the 2%/1 mm criterion could identify MLC misalignment errors greater than 0.5 mm. Although the 1%/1 mm criterion appeared capable of identifying errors greater than 0.25 mm, the original error-free plan may also fall below the 80% acceptance threshold, indicating reduced specificity and the possibility of false-positive classification (Figure 3d).

In the Octavius 4D results, the gamma pass rates of the original plan were found to be below the 90% pass rate for all criteria except for the 3%/3 mm gamma criterion. If the tolerance level is accepted as 90%, errors larger than 0.25 mm are found to be detectable for all gamma criteria except for the 3%/3 mm criterion. The 2%/1 mm criterion was also found to be the second most sensitive gamma criteria in the Octavius 4D results. At the 80% tolerance level, the 1%/2 mm, 2%/1 mm, and 1%/1 mm gamma criteria were found to successfully detect MLC misalignment errors after 0.25 mm (Figure 3e).

A general evaluation of the graphs shown in Figure 3 demonstrates that the 1%/1 mm and 2%/1 mm criteria are the most effective for identifying small systematic MLC aperture widening errors. However, reducing the gamma acceptance percentage to 80% may cause the error to be overlooked. The differences were observed more clearly at values in the higher passing-rate range; particularly between 90 and 95%, the detectable MLC position error value can be measured up to 0.5 mm depending on the gamma criteria. This indicates how sensitive gamma analysis is in error detection and how effective these systems are in different error scenarios. However, the 1%/1 gamma criterion is a stricter threshold and accounts for other errors not related to the MLC, which is why it is not recommended as a gamma criterion.

The comparative evaluation of the dosimetric systems presented in Figure 3 indicates that the observed differences in gamma sensitivity are strongly influenced by the dosimetric system characteristics of each platform, including detector resolution, dose reconstruction algorithm, and evaluation method. Systems such as EPIQA and COMPASS, which employ high-resolution EPID panels or dense detector matrices, demonstrate a more pronounced reduction in gamma passing rates as the magnitude of the imposed MLC error increases. This behavior reflects an enhanced ability to capture subtle geometric deviations within highly modulated VMAT fields. In contrast, lower-resolution systems such as ArcCHECK 3DVH and the Octavius 4D ion-chamber array exhibit comparatively smoother gamma responses, suggesting a reduced sensitivity to small MLC aperture widening errors. These findings emphasize that gamma performance is not solely governed by the selected gamma criteria but is also significantly affected by the physical and algorithmic properties of the dosimetric system.

Additionally, Table 1 lists the slope values derived from the regression lines describing the relationship between the passing rates and the magnitudes of MLC deviations. A greater absolute gradient indicates a higher degree of sensitivity in the gamma analysis. Accordingly, the most sensitive gamma criterion was determined to be 1%/1 mm for EPIQA and 2%/1 mm for COMPASS measurements.

The magnitude of the slope values derived from Table 1 also provides clinically relevant insight into the sensitivity of each dosimetry system. Larger absolute slopes—such as those observed for EPIQA under the 1%/1 mm criterion and for COMPASS under the 2%/1 mm criterion—indicate a rapid decline in gamma passing rates with increasing MLC aperture widening, thereby reflecting a high detectability for small geometric deviations. This characteristic is particularly important for SBRT, where steep dose gradients amplify the dosimetric impact of submillimeter errors. Conversely, systems with lower slope values, including ArcCHECK 3DVH and Octavius 4D, exhibit a more gradual change in gamma passing rates, suggesting that certain clinically relevant deviations may remain insufficiently reflected in the gamma analysis. These results highlight the need to consider detector-specific response characteristics when interpreting gamma-based QA metrics. Under the 1%/2 mm criterion, the highest sensitivity was recorded for COMPASS (slope: −14.99%/mm). This system demonstrated a steeper decline in gamma passing rate as MLC aperture widening increased, indicating stronger responsiveness to small leaf deviations compared to the other detectors. At the 2%/1 mm criterion the difference between systems became more apparent. COMPASS again showed the largest absolute slope (–20.8%/mm), suggesting a consistent ability to identify even subtle MLC shifts. In contrast, ArcCHECK 3DVH and Octavius 4D exhibited smaller gradients, implying a reduced sensitivity under the same conditions. When the most stringent criterion (1%/1 mm) was applied, EPIQA achieved the highest sensitivity with a slope of −21.8 %/mm. The rapid reduction in gamma passing rate with increasing MLC aperture widening reflected the system’s high sensitivity to small MLC aperture width errors. Across all configurations, a general trend was observed: stricter gamma criteria produced larger absolute slopes for all systems, revealing enhanced geometric deviation detection. Among the tested configurations, EPIQA and COMPASS exhibited the steepest decreases in gamma passing rate with increasing MLC aperture widening, indicating high sensitivity based on regression slope analysis. However, this finding should be interpreted separately from specificity, as COMPASS also demonstrated relatively low gamma passing rates for the error-free plans.

Global gamma passing rates for error-free (MLC = 0 mm) and intentional error (MLC = 1 mm) plans under different gamma criteria are shown in Figure 4a. In the bar chart, the lowest pass rates were observed with the Octavius 4D dosimetry system in both error-free and MLC 1 mm erroneous treatment plans. For the 2%/1 mm and 1%/1 mm gamma criteria, the lowest gamma pass rates with a 1 mm MLC misalignment were found with the COMPASS dosimetry system, while the highest gamma pass rates were found with the EPIQA dosimetry system. In the COMPASS dosimetry system, the gamma passing rate was found to be 39.9% lower than the 1%/2 mm gamma criterion, while in other dosimetry systems, it was found to be lower by 18.13% for EPIQA, 19.4% for SNCPATIENT, 10.95% for 3DVH, and 5.4% for Octavius 4D, respectively.

The variation in the magnitude of gamma differences between error-free and erroneous plans shown in Figure 4a demonstrates that each system responds distinctively to intentional MLC deviations. Although EPIQA consistently yields the highest absolute passing rates, the relatively smaller difference between 0 mm and 1 mm plans suggests a less pronounced response to geometric perturbations. This behavior may be associated with EPID-specific factors such as backscatter effects or smoothing processes in portal dose prediction. In contrast, COMPASS exhibits lower overall passing rates but a markedly larger drop in the presence of a 1 mm MLC error, highlighting its greater responsiveness to systematic deviations in leaf positioning and MU modulation. This differing behavior between systems underscores the importance of understanding how detector architecture and reconstruction methodology influence error detectability. Overall, these results suggest that while EPIQA achieves superior passing rates, its relatively smaller difference between error-free and 1 mm misalignment plans may indicate limited error detectability. Conversely, COMPASS shows lower absolute passing rates but a larger decline when errors are introduced, demonstrating higher responsiveness to geometric deviations.

In local passing rates, the EPIQA dosimetric system was found to have the highest gamma pass rates across all gamma criteria, just as it does in global pass rates, compared to all other dosimetric systems (Figure 4b) Due to a systematic error in the COMPASS dosimetric system, local pass rates could not be obtained. Among all dosimetric systems other than EPIQA, the 2%/1 mm gamma criterion had the lowest gamma pass rates after the 1%/1 mm gamma criterion. The discrepancy between global and local gamma passing rates, especially under stricter criteria such as 1%/1 mm, reflects the distinct dose-normalization approaches of the two methods. Local gamma, which normalizes dose differences to the calculated local dose at each point, inherently amplifies deviations occurring in high-gradient regions, making it more sensitive to geometric perturbations characteristic of SBRT. System-specific detector configurations and software-based dose reconstruction processes may influence error-detection sensitivity, particularly when global gamma evaluation is used [4,18]. In the present study, this effect was particularly evident for the 3DVH and Octavius 4D systems. Consequently, the selection of global versus local gamma has a direct impact on the sensitivity of QA analysis sensitivity, and local gamma may provide a more clinically meaningful assessment in high-gradient treatment techniques.

The discrepancy between global and local gamma evaluations became most pronounced under the strictest criteria (1%/1 mm) particularly for 3DVH and Octavius 4D. Taken together, the global and local analyses reveal complementary aspects of system performance. Global analysis provides a broad overview of agreement between measured and calculated doses, while local analysis highlights fine spatial discrepancies, particularly in high-dose-gradient regions. The significant divergence between global and local results under stricter criteria (especially 1%/1 mm) underscores how the choice of analysis approach can critically influence QA outcomes. In SBRT VMAT plans, where sub-millimeter precision is required, local gamma evaluation may offer a more realistic representation of system accuracy and error sensitivity.

To further investigate the relationship between the global and local gamma evaluation methods, Pearson correlation analysis was performed using the entire dataset to determine the level of agreement between the two approaches under different gamma criteria for each dosimetric system. The resulting Pearson correlation coefficients and corresponding *p*-values are presented in Table 2.

Except for Figure 4b, all gamma passing rates plotted in the figures and listed in the tables were obtained using global gamma analysis. Figure 4b presents local gamma passing rates. Table 2 presents the Pearson correlation coefficients and corresponding *p*-values for the relationship between global and local gamma passing rates, calculated using the entire dataset. High correlations observed in EPIQA and Octavius 4D for most gamma criteria indicate that both evaluation methods respond in a similar manner to dose deviations. However, the weak or absent correlations reported for SNCPATIENT and 3DVH under certain criteria suggest that global gamma may fail to adequately capture spatial variations that are more clearly reflected in local analysis. This divergence is particularly relevant in SBRT treatments, where deviations in high-gradient regions carry greater clinical significance. These findings reinforce the notion that gamma evaluation should not rely solely on global criteria but should also incorporate local analysis, particularly when submillimeter geometric accuracy is required. For EPIQA measurements, the strongest correlation between global and local gamma passing rates was observed using the 2%1 mm gamma criterion (r = 0.925, *p* < 0.001). With all gamma criteria except the 1%/2 mm criterion, moderate and strong correlations (r > 0.5, *p* < 0.001) were observed between the global and local gamma passing rates. For SNCPATIENT measurements, the highest correlation between global and local gamma passing rates was observed with the 1%/2 mm gamma criterion (r = 0.939, *p* < 0.001). The strongest correlations were found with both the 1%/2 mm and 1%/1 mm criteria. No correlation was observed between global and local gamma passing rates for the 3%/3 mm, 3%/2 mm, and 2%/1 mm criteria. With the 2%/2 mm gamma criterion, a moderate correlation was observed between global and local gamma passing rates (r = 0.580, *p* = 0.002). For 3DVH measurements, the highest correlation between global and local gamma passing rates was observed with the 1%/2 mm gamma criterion (r = 0.689, *p* < 0.001). Moderate correlations were observed between global and local gamma passing rates for the 3%/3 mm, 2%/2 mm, and 1%/1 mm criteria (*p* < 0.005). No correlation was observed between global and local gamma passing rates for the 3%/2 mm and 2%/1 mm criteria (Table 2). For Octavius 4D measurements, the highest correlation between global and local gamma passing rates was observed with the 1%/2 mm gamma criterion (r = 0.917, *p* < 0.001). Moderate to high correlations were observed between global and local gamma passing rates for all gamma criteria (*p* < 0.001) (Table 2).

### Sensitivity and Specificity

**Figure 5:**
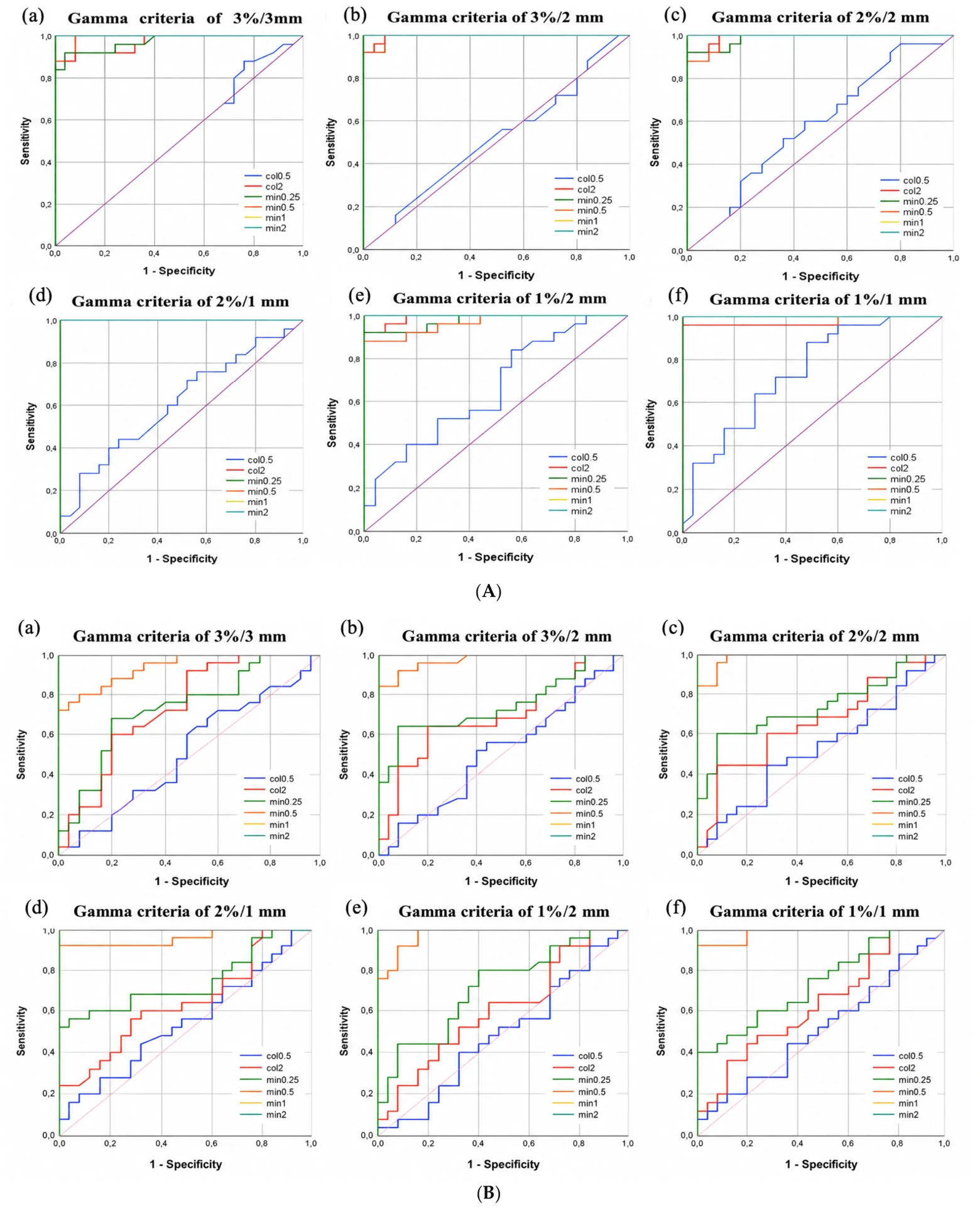

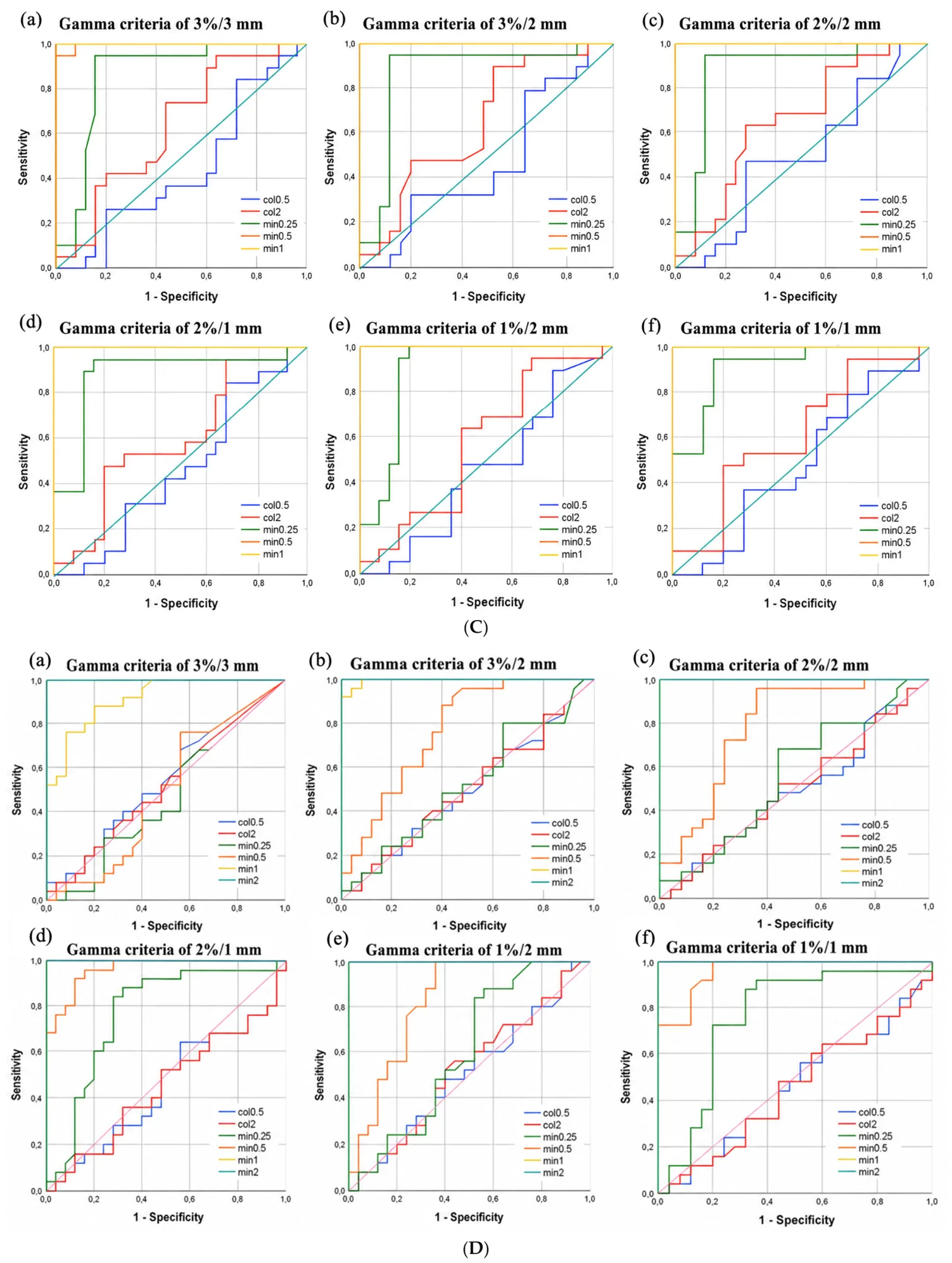

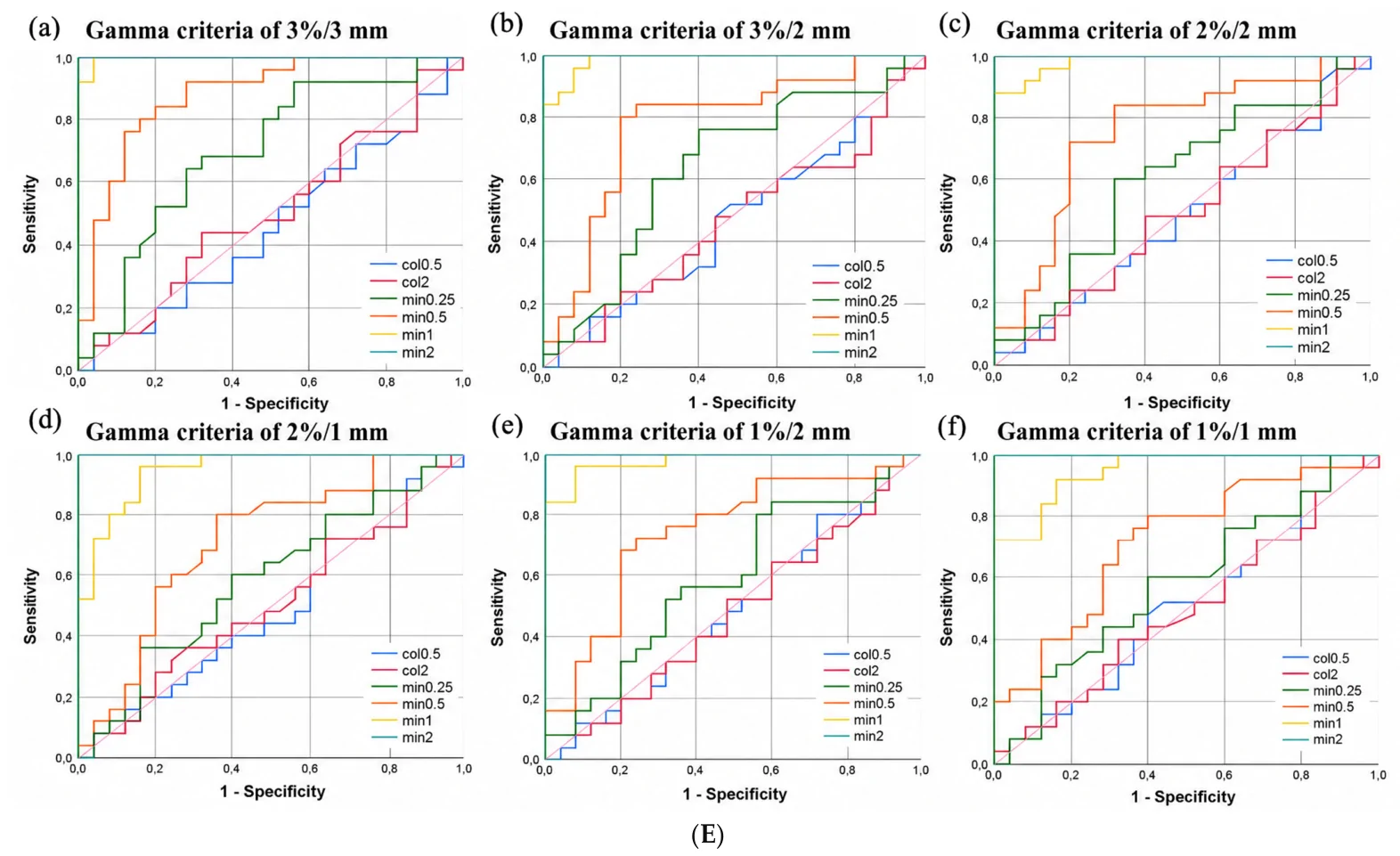
(**A**) Receiver operating characteristic curve of various gamma criteria when using EPIQA. Receiver operating characteristic (ROC) curves of MLC errors of 0.25, 0.5, 1 and 2 mm, as well as collimator errors of 0.5 and 2 degrees are shown. The ROC curves of gamma criteria of 3%/3 mm (**a**), 3%/2 mm (**b**), 2%/2 mm (**c**), 2%/1 mm (**d**), 1%/2 mm (**e**) and 1%/1 mm (**f**) when using EPIQA. (**B**) Receiver operating characteristic curve of various gamma criteria when using SNCPATIENT. Receiver operating characteristic (ROC) curves of MLC errors of 0.25, 0.5, 1 and 2 mm, as well as collimator errors of 0.5 and 2 degrees are shown. The ROC curves of gamma criteria of 3%/3 mm (**a**), 3%/2 mm (**b**), 2%/2 mm (**c**), 2%/1 mm (**d**), 1%/2 mm (**e**) and 1%/1 mm (**f**) when using SNCPATIENT. (**C**) Receiver operating characteristic curve of various gamma criteria when using ArcCHECK-3DVH. Receiver operating characteristic (ROC) curves of MLC errors of 0.25, 0.5, and 1 mm, as well as collimator errors of 0.5 and 2 degrees are shown. The ROC curves of gamma criteria of 3%/3 mm (**a**), 3%/2 mm (**b**), 2%/2 mm (**c**), 2%/1 mm (**d**), 1%/2 mm (**e**), and 1%/1 mm (**f**) when using ArcCHECK-3DVH. (**D**) Receiver operating characteristic curve of various gamma criteria when using COMPASS. Receiver operating characteristic (ROC) curves of MLC errors of 0.25, 0.5, 1 and 2 mm, as well as collimator errors of 0.5 and 2 degrees are shown. The ROC curves of gamma criteria of 3%/3 mm (**a**), 3%/2 mm (**b**), 2%/2 mm (**c**), 2%/1 mm (**d**), 1%/2 mm (**e**) and 1%/1 mm (**f**) when using COMPASS. (**E**) Receiver operating characteristic curve of various gamma criteria when using Octavius 4D. Receiver operating characteristic (ROC) curves of MLC errors of 0.25, 0.5, 1 and 2 mm, as well as collimator errors of 0.5 and 2 degrees are shown. The ROC curves of gamma criteria of 3%/3 mm (**a**), 3%/2 mm (**b**), 2%/2 mm (**c**), 2%/1 mm (**d**), 1%/2 mm (**e**) and 1%/1 mm (**f**) when using Octavius 4D.

ROC curves and AUC values were used to evaluate the sensitivity and specificity of different gamma criteria across the dosimetric systems. The ROC curves and the AUC values are shown in Figure 5A–E and Table 3a–f, respectively, for each gamma criteria at different magnitudes of MLC aperture widening and collimator rotation errors across the different dosimetric systems. For all gamma criteria and dosimetric systems, 0.50 Col rotation showed the worst performance. Between the dosimetric systems, EPIQA showed the best performance for the all-gamma criteria. The performances of Octavius 4D and Compass systems are relatively low compared to the others. Generally, stricter gamma criteria result in lower AUC values. The 1%/1 mm Gamma criterion has the highest results for all type of errors and dosimetric systems. Both for the collimator rotation and MLC aperture widening errors, the larger systematic error value results with higher AUC values.

In the EPIQA measurements, the AUC values obtained from ROC analysis for 0.25 mm, 0.5 mm, and 1 mm MLC errors were 1.0 for all cases under the 2%/1 mm γ criterion. Under the 1%/2 mm criteria, the AUC values were 0.976, 0.965, and 1.0, respectively, while for the 1%/1 mm criterion, they were 1.0, 0.976, and 1.0. For SNCPATIENT, AUC values for 0.25 mm, 0.5 mm, and 1 mm MLC errors were 0.746, 0.958, and 1.0 for the 2%/1 mm criterion; 0.719, 0.976, and 1.0 for the 1%/2 mm criterion; and 0.744, 0.984, and 1.0 for the 1%/1 mm criterion. For ArcCHECK 3DVH, the corresponding AUC values for 0.25 mm, 0.5 mm, and 1 mm MLC errors were 0.88, 1.0, and 1.0 for the 2%/1 mm criterion; 0.886, 1.0, and 1.0 for the 1%/2 mm criterion; and 0.914, 1.0, and 1.0 for the 1%/1 mm criterion. Using the COMPASS system, AUC values for the same error magnitudes were 0.77, 0.962, and 1.0 under 2%/1 mm; 0.604, 0.826, and 1.0 under 1%/2 mm; and 0.762, 0.959, and 1.0 under 1%/1 mm. For OCTAVIUS 4D, the ROC analysis yielded AUC values of 0.586, 0.714, and 0.949 for 2%/1 mm; 0.589, 0.745, and 0.978 for 1%/2 mm; and 0.581, 0.716, and 0.949 for 1%/1 mm γ criterion, respectively. Overall, nearly perfect sensitivity and specificity were achieved in detecting large (≈2 mm) MLC aperture widening errors across all systems. However, both sensitivity and specificity declined as the magnitude of these errors decreased to 1 mm and below demonstrate that the high-resolution EPID-based EPIQA system can detect small, systematic MLC positional errors more reliably compared to systems with relatively lower detector resolution (ArcCHECK, COMPASS, and Octavius 4D). This observation is also supported by recent investigations demonstrating that detector spatial resolution and detector design substantially influence the ability to detect subtle delivery errors in stereotactic radiotherapy. Consequently, systems with coarser spatial sampling may fail to identify subtle MLC deviations that can still be clinically relevant.

The ROC and AUC findings demonstrate that error detectability varies considerably between systems and gamma criteria. The consistently high AUC values observed for EPIQA across multiple error magnitudes reflect its superior sensitivity in distinguishing error-free deliveries from those with systematic deviations. Systems employing lower spatial resolution, such as Octavius 4D, reveal substantially lower AUC values for small-magnitude errors, indicating reduced discriminative capacity. Additionally, gamma criteria with broader tolerances, including 3%/3 mm, exhibit lower AUC values across all systems, supporting the conclusion that such criteria are insufficiently sensitive for SBRT VMAT quality assurance. Stricter criteria—particularly 1%/1 mm and 2%/1 mm—consistently demonstrate enhanced error detection, reinforcing the necessity of employing more stringent gamma thresholds in high-precision radiotherapy.

Figure 5C shows eceiver operating characteristic (ROC) curves obtained using the 3DVH system. ROC curves for MLC aperture widening errors of 0.25, 0.5, and 1 mm, as well as collimator rotation errors of 0.5° and 2°, are presented. Panels (a–f) correspond to the gamma criteria of 3%/3 mm, 3%/2 mm, 2%/2 mm, 2%/1 mm, 1%/2 mm, and 1%/1 mm, respectively. The 2-mm MLC aperture widening error was excluded from the ROC analysis because the extremely low gamma passing rates under the strictest gamma criteria resulted in insufficient variability for reliable AUC calculation.

Figure 5A–E present the ROC curves obtained using the EPIQA, SNC Patient, 3DVH, COMPASS, and Octavius 4D systems under various gamma criteria. Each detector was evaluated for its ability to distinguish error-free plans from those including systematic MLC aperture widening errors of 0.25, 0.5, 1, and 2 mm, as well as collimator rotation errors of 0.5° and 2°. The deviation of each ROC curve from the diagonal reference line indicates the discriminative capability of the detector–gamma-criteria combination. Among all evaluated systems, EPIQA demonstrated the strongest overall discriminative performance. ROC curves for gamma criteria of 2%/2 mm, 2%/1 mm, and 1%/1 mm showed pronounced left-upper quadrant clustering for 0.5–1 mm MLC errors, with AUC values typically between 0.90 and 1.00. Even under more lenient criteria (3%/3 mm and 3%/2 mm), EPIQA maintained high sensitivity for errors ≥ 0.5 mm. These findings demonstrate that the EPID-based EPIQA system can detect small, systematic MLC aperture widening errors more reliably than the other evaluated dosimetric systems. This superior performance reflects its dosimetric system characteristics, including detector resolution, dose reconstruction algorithm, and evaluation method. Consequently, dosimetric systems with different characteristics may fail to identify subtle systematic MLC aperture widening errors (Figure 5A). SNCPATIENT exhibited a more moderate ROC performance compared with EPIQA. Under 3%/3 mm and 3%/2 mm criteria, ROC curves were positioned close to the diagonal line, indicating limited discriminative strength and suggesting that these gamma thresholds are too permissive for meaningful error detection with ArcCHECK. Performance improved under 2%/2 mm and 2%/1 mm, but sensitivity remained insufficient for identifying 0.25 mm and 0.5 mm deviations. Under the strictest 1%/1 mm criterion, AUC values increased to the 0.70–0.85 range for larger errors, although small deviations continued to be poorly detected. Overall, SNC Patient demonstrated reduced sensitivity to small MLC errors, with acceptable performance only for more pronounced deviations (Figure 5B). 3DVH, based on volumetric dose reconstruction, produced noticeably stronger ROCs than ArcCHECK raw measurements. ROC curves became more separated from the diagonal under 2%/2 mm, 2%/1 mm, and 1%/1 mm criteria, particularly for 0.5–1 mm MLC errors, where AUC values commonly exceeded 0.90. Although the 3% criterion provided limited sensitivity for 0.25 mm deviations, they remained effective for ≥1 mm errors. These results indicate that the dose-reconstruction approach in 3DVH enhances the detectability of clinically meaningful MLC aperture widening errors compared with 2D detector surface-dose analysis (Figure 5C). COMPASS showed a more heterogeneous ROC performance. Under 3%/3 mm and 3%/2 mm criteria, ROC curves were clustered near the diagonal, indicating weak discriminative ability and suggesting substantial masking of small errors masking. Moderate improvements were observed under 2%/2 mm and 2%/1 mm criteria, although overall sensitivity remained limited for deviations ≤ 0.5 mm. Even under the strictest 1%/1 mm gamma criterion, AUC values generally lagged those of EPIQA and 3DVH, reflecting lower responsiveness to subtle MLC shifts. These findings suggest that COMPASS requires tight criteria to detect meaningful deviations yet still demonstrates comparatively lower sensitivity than other systems (Figure 5D). Octavius 4D demonstrated intermediate performance between EPIQA/3DVH and SNC Patient/COMPASS. Under lenient criteria (3%/3 mm and 3%/2 mm), ROC curves were moderately displaced from the diagonal, showing partial discriminative strength. Sensitivity improved markedly under 2%/1 mm, 1%/2 mm, and 1%/1 mm criteria, for which AUC values approached 0.85–0.95 for 0.5–1 mm error. These trends suggest that Octavius 4D has solid capability for detecting moderate-to-large MLC aperture widening errors, while stricter gamma thresholds are required to maximize performance for smaller MLC aperture widening errors (Figure 5E).

Collectively, the ROC analysis demonstrates that EPIQA provides the highest and most consistent sensitivity across all gamma criteria, particularly in detecting small MLC aperture widening errors. 3DVH offers strong discriminative capability, particularly when using tighter gamma thresholds. Octavius 4D performs reliably for moderate error magnitudes with improved performance under strict criteria. SNC Patient and COMPASS show limited sensitivity to small errors, with performance improving only for larger deviations or under strict gamma criteria. Overall, the results clearly indicate that the combination of detector choice and gamma criteria substantially influences the ability to detect MLC aperture widening and collimator rotation errors, with reconstructed-dose and high-resolution EPID-based systems outperforming detector-array systems in detecting small-magnitude errors.

Taken together, the gamma analysis, slope evaluation, correlation assessment, and ROC-based sensitivity analysis show clear differences in the error-detection capabilities of the examined dosimetry systems. High-resolution EPID-based systems such as EPIQA exhibit superior sensitivity to submillimeter deviations, whereas diode-array-based systems demonstrate varying degrees of responsiveness depending on detector geometry and reconstruction methodology. The findings also confirm that stricter gamma criteria markedly improve error detectability and that conventional criteria such as 3%/3 mm are inadequate for SBRT VMAT verification. Overall, the results highlight the critical importance of selecting both the appropriate detector system and gamma criteria to ensure reliable identification of clinically relevant MLC aperture widening errors in high-precision radiotherapy.

## 4. Discussion

Patient-specific QA aims to ensure that all parameters are correctly transferred from the planning system to the treatment device, ensuring the measured and expected dose distributions from the TPS are closely aligned. Due to the complexity of VMAT planning and delivery, patient-specific QA is recommended for each new patient before treatment. This ensures that any major errors, such as data transfer issues or incorrect beam energy, can be detected. This is typically achieved by comparing measurements from ion chambers, film, and detector arrays (ion chambers or diodes) with the planned calculations. Additionally, it is effective in identifying subtle errors, such as MLC aperture width inaccuracies or TPS calculation mistakes, which can influence dose accuracy. The gamma index (γ), which combines percentage dose difference (%diff) and distance to agreement (DTA), is a widely used metric for this comparison [21,22,23].

Our main objective was to investigate the influence of dosimetric system characteristics on gamma index passing rates across different dosimetric systems. This objective was achieved by evaluating the sensitivity of different gamma criteria to systematic MLC aperture widening errors across multiple dosimetric systems. Six gamma criteria (3%/3 mm, 3%/2 mm, 2%/2 mm, 2%/1 mm, 1%/2 mm, and 1%/1 mm) were applied with both global and local tolerances for ArcCHECK, IBA MATRIXX, and EPIQA. However, only global gamma analysis was evaluated for the COMPASS system. It also explored the effect of dosimetric system characteristics on gamma passing rates across different dosimetric systems. Gamma analysis is a routine method in busy clinics for patient quality assurance (QA), which makes it important to understand its limitations and sensitivity of the gamma method and the dosimetry system being used. The study also aimed to compare the sensitivity of different gamma passing rate values to various delivery errors and to determine an appropriate gamma analysis method for detecting specific errors that may arise in clinical practice. It is crucial to select suitable gamma criteria and thresholds based on the complexity of the treatment plan technique, the detector system’s limitations, and the QA technique being used.

The decrease in local gamma passing rates due to increased linac-based errors was more pronounced than in global gamma passing rates, as local gamma analysis is stricter in evaluating each point individually compared to global gamma analysis. Our primary objective was to examine the impact of dosimetric system characteristics on gamma index passing rates across different dosimetric systems. This was accomplished by evaluating the sensitivity of different gamma acceptance criteria under simulated systematic MLC errors in VMAT plans and assessing sensitivity and performance across EPID, planar, and cylindrical arrays. The findings indicate that the 2%/1 mm gamma index criterion is the most sensitive for detecting systematic MLC errors.

Comparison of the global and local gamma passing rates showed that local gamma analysis generally produced a more pronounced reduction in passing rates following the introduction of MLC aperture-widening errors among the dosimetric systems for which local results were available. However, the linear regression analysis and the slopes of the best-fit lines were based exclusively on global gamma passing rates. Our results suggest that either the 2%/1 mm or 1%/1 mm criteria provide the highest sensitivity for gamma index analysis, depending on the dosimetric system characteristics and the magnitude of the introduced MLC aperture widening errors. In gamma comparisons, the appropriate normalization method remains a topic of debate due to the strengths and weaknesses of both local and global normalization methods. Global normalization uses the maximum dose of the calculated dose distribution, while local normalization uses the calculated dose at the evaluated pixel. In a study using planar devices, it was shown that global normalization can mask some errors in low-dose regions. Similarly, using global normalization, which normalizes error percentages based on the maximum dose in the plan or field, may conceal significant, small errors in critical structures where organ tolerance is already near its limit. Therefore, the choice between local and global normalization should be made based on clinical priorities: maximizing sensitivity to small systematic errors (favoring local normalization) versus minimizing noise-induced false detections in heterogeneous dose regions (favoring global normalization).

Our study demonstrated that dosimetric system characteristics significantly influence gamma sensitivity. High-resolution systems such as EPIQA and COMPASS exhibited sharper declines in passing rates and higher slope magnitudes with increasing MLC deviations. These differences are attributable not only to dosimetric system characteristics but also to fundamental differences in dose reconstruction methodology. EPIQA performs detector-level two-dimensional gamma analysis without reconstructing the dose distribution within the patient anatomy. ArcCHECK 3DVH estimates the three-dimensional patient dose distribution using a measurement-guided dose reconstruction algorithm based on measured deviations from the planned dose distribution. In contrast, COMPASS reconstructs the full three-dimensional dose distribution within the patient anatomy using an independent beam model, whereas the Octavius 4D system reconstructs the three-dimensional dose distribution within the phantom. These methodological differences should be considered when interpreting differences in gamma analysis performance among these systems should be interpreted in the context of both their detector characteristics and their underlying dose reconstruction methodologies. Our study demonstrated that dosimetric system characteristics significantly influence gamma sensitivity. High-resolution systems such as EPIQA and COMPASS exhibited sharper declines in passing rates and higher slope magnitudes with increasing MLC deviations. In particular, the superior sensitivity observed with the EPID-based EPIQA system can be attributed, at least in part, to its high spatial resolution, which facilitates the detection of small systematic MLC aperture-widening errors compared with lower-resolution detector arrays. These differences are attributable not only to detector resolution but also to fundamental differences in dose reconstruction and evaluation methodologies. These results are consistent with the observations of Chun et al. [24], who demonstrated that dosimetric system characteristics, including detector resolution, influence the ability of the gamma metric to identify delivery errors. Likewise, Woon et al. [25] reported that dosimetric systems with relatively coarse detector resolution exhibited lower sensitivity for detecting small delivery errors—such as the ArcCHECK system—may not reliably detect subtle yet clinically important deviations. In parallel, Fredh et al. [2] also highlighted substantial variation between different QA devices, noting pronounced discrepancies in the performance of EPIQA, Octavius, Delta4, and COMPASS when subjected to similar error conditions. Letourneau et al. [26] validated the ArcCHECK system for VMAT delivery verification, although most of their assessments relied on the 3%/2 mm gamma criterion, which inherently limited the device’s ability to reveal subtle geometric deviations. In a multi-detector analysis of 50 VMAT plans, Masi et al. [5] recommended adopting the 3%/2 mm criterion instead of the more permissive 3%/3 mm threshold, emphasizing improved sensitivity to clinically meaningful discrepancies. Additionally, both Spezi et al. [27] and Syamkumar et al. [28] demonstrated that 1-mm intentional MLC aperture widening could be detected using the PTW seven29 array during beam-by-beam IMRT verification. However, these evaluations were conducted primarily within IMRT or conventional VMAT frameworks, and the applicability of their criteria to SBRT—where geometric precision is significantly more critical—remains uncertain.

Our results indicate that widely used criteria such as 3%/3 mm lack sufficient sensitivity to identify systematic MLC deviations of 1 mm or smaller in SBRT VMAT deliveries. This observation is consistent with the work of Heilemann et al. [3] and Yan et al. [29], both of whom reported that the 2%/2 mm criterion provide superior sensitivity to geometric inaccuracies. Within our dataset, the 2%/1 mm threshold demonstrated the strongest and most consistent performance across all detector systems, surpassing both 3%/2 mm and 2%/2 mm in its ability to detect subtle systematic leaf-position errors. Although stricter gamma criteria enhance detection sensitivity, the 1%/1 mm threshold is not feasible for routine clinical use. Consistent with these findings, our analysis also revealed pronounced instability and markedly reduced passing rates—even in error-free plans—particularly in dosimetric systems with characteristics that reduce sensitivity to subtle delivery errors. In addition, the observed instability of the 1%/1 mm criterion may be partly attributable to the 1 mm dose-calculation grid, which is not appreciably finer than the 1 mm DTA search distance. Under these conditions, interpolation-related uncertainty may contribute to variability in gamma analysis. Therefore, this should be considered as a methodological limitation when interpreting results obtained with very strict gamma criteria.

As highlighted by Heilemann et al. and Syamkumar et al. [3,28], applying such a criterion tends to magnify statistical noise and accentuate detector-specific limitations, ultimately providing minimal added diagnostic value. Consistent with these findings, our analysis also revealed pronounced instability and markedly reduced passing rates—even in error-free plans—particularly in dosimetric systems with characteristics that reduce sensitivity to subtle delivery errors. The relatively poor detectability of the 0.5° collimator rotation observed in this study can be explained by the limited geometric displacement produced around the isocenter in single-target lung SBRT VMAT plans. Because the rotational effect increases with the distance from the isocenter, small collimator rotations have only a limited dosimetric impact in centrally located single-target treatments. In contrast, single-isocenter multi-target stereotactic treatments, where lesions are distributed at varying distances from the isocenter, are expected to be considerably more sensitive to collimator rotation errors. Therefore, the findings of the present study regarding collimator rotation should be interpreted within the context of single-target SBRT VMAT.

Detector design also played a notable role in the divergence observed between global and local gamma evaluations. In agreement with findings from Yu et al. [30] and Letourneau et al. [26], local gamma analysis demonstrated heightened sensitivity in steep dose-gradient regions and showed more pronounced reductions as error magnitude increased, whereas global gamma metrics frequently obscured these deviations. Reconstruction-based platforms, including 3DVH and Octavius 4D, exhibited the greatest discrepancies between global and local gamma results, likely attributable to their inherent reliance on smoothing, interpolation, and model-based dose reconstruction algorithms. A limitation of the present study is that the clinical dosimetric impact of the introduced MLC and collimator errors was not evaluated using dose–volume histogram (DVH) analysis. The primary objective of this work was to investigate the sensitivity of different dosimetric systems and gamma criteria for detecting intentional mechanical errors. Although gamma analysis provides valuable information regarding error detectability, future studies incorporating DVH comparisons between the original and modified plans would further clarify the clinical significance of the detected errors. The findings of this study should be interpreted within the context of the simulated error model. The present analysis focused exclusively on systematic symmetric MLC gap-opening errors representing calibration-related deviations. In contrast, asymmetric single-bank, individual leaf, or stochastic leaf-position errors may produce more localized dose perturbations and partial error cancellation, potentially resulting in different gamma index sensitivity and ROC performance. Future investigations should evaluate these clinically relevant error patterns to determine whether the observed diagnostic performance remains applicable under more complex error conditions.

ROC and AUC analyses also supported the detector-dependent differences observed in our gamma results. For larger MLC deviations (≥1 mm), all systems produced AUC values approaching unity, consistent with the findings of Fredh et al. [2] and Vieillevigne et al. [31]. In contrast, smaller systematic errors (0.25–0.5 mm) revealed marked performance disparities among the dosimetric systems: EPIQA consistently maintained high AUC scores, whereas ArcCHECK and Octavius 4D demonstrated reduced sensitivity, underscoring the influence of dosimetric system characteristics—including detector resolution, dose reconstruction algorithm, and evaluation method—on the ability to detect clinically relevant errors [32]. An independent dose–volume histogram (DVH) analysis correlating the gamma results with dose–volume endpoints was not performed. Although the systematic MLC aperture-widening errors investigated in this study would be expected to produce a coherent DVH shift rather than a randomly distributed discrepancy, Nelms et al. [33] demonstrated that an apparently acceptable gamma passing rate may nevertheless conceal clinically relevant DVH deviations. Therefore, establishing the quantitative relationship between the recommended gamma criteria and PTV/OAR dose–volume changes remain an important direction for future work. Although ROC/AUC analysis provides a robust framework for comparing detector performance, the selection of an optimal gamma passing-rate threshold for clinical implementation remains dependent on the specific clinical application. Recent studies have similarly highlighted the value of ROC-based analyses for optimizing patient-specific QA decision thresholds in stereotactic radiotherapy [34]. Future studies may investigate threshold selection using approaches such as the Youden index or predefined sensitivity and specificity targets, together with detector characteristics and treatment complexity.

Several limitations should be acknowledged. First, only systematic, symmetric aperture-widening MLC errors applied equally to each leaf bank were modeled; because such widening couples a geometric (distance-to-agreement) change with a global output/field-size dose increase, it perturbs the dosimetric and geometric components of the gamma criteria simultaneously, and the criteria- and detector-dependent sensitivities reported here may not transfer directly to asymmetric single-bank, aperture-shift, or random per-leaf errors, which warrant dedicated investigation. Second, an independent dose–volume histogram (DVH) analysis correlating the gamma results with dose–volume endpoints was not performed; although the systematic aperture-widening error studied here would be expected to produce a coherent DVH shift rather than a randomly distributed discrepancy—precisely the situation in which, as Nelms et al. [33] noted, an apparently acceptable gamma passing rate may nonetheless conceal a clinically relevant DVH deviation—establishing the quantitative relationship between the recommended gamma criteria and PTV/OAR dose–volume changes remains an important direction for future work. Third, a 10% low-dose threshold was applied throughout; in SBRT some organs at risk may receive doses in the 5–10% range, and for error types that redistribute dose into low-dose regions a lower threshold could alter error detectability. These considerations reinforce the recommendation that gamma-based QA be complemented by DVH and clinical-endpoint evaluation, consistent with AAPM TG-218. Another limitation of this study is that the PTW Octavius 4D system was used with the Seven29 detector array rather than the newer Octavius 1500 (XDR) array, which provides improved dosimetric system characteristics, including higher spatial resolution, and may therefore offer greater sensitivity for detecting delivery errors in highly modulated VMAT SBRT plans. Therefore, the present findings obtained with the Seven29 array should not be directly generalized to newer detector configurations.

Although this study was conducted using lung SBRT VMAT plans, the proposed methodology—including intentional systematic error insertion, multi-criteria global/local gamma analysis, and ROC/AUC-based performance evaluation—may also be applied to other radiotherapy techniques, such as conventional VMAT, IMRT, cranial SRS, and spine SBRT. However, the quantitative findings of this study, particularly the superior performance of the 2%/1 mm gamma criterion, are specific to the high-dose-gradient and small-target characteristics of lung SBRT VMAT. Therefore, the optimal gamma criteria may differ for other treatment sites and delivery techniques, and further validation is warranted before generalizing these findings.

## 5. Conclusions

This study demonstrates that gamma analysis performance in SBRT VMAT is strongly dependent on dosimetric system characteristics as well as the selected gamma criteria. EPIQA demonstrated superior overall diagnostic performance for detecting submillimeter MLC deviations, whereas COMPASS exhibited higher sensitivity but lower specificity. In contrast, ArcCHECK and Octavius 4D showed reduced responsiveness, reflecting differences in dosimetric system characteristics. Among the criteria evaluated, 2%/1 mm provided the most robust and stable error detection, while traditional 3%/3 mm thresholds were insufficient for identifying clinically relevant MLC aperture widening errors. Conversely, the 1%/1 mm criterion introduced instability without improving clinical detection capability.

Our findings highlight that gamma criteria and QA strategies must be tailored to detector-specific capabilities and the geometric demands of SBRT. High-gradient, small-target radiotherapy requires stricter gamma thresholds and higher-resolution detectors to ensure accurate error detection. These results support transitioning from universal gamma criteria toward technique-specific, device-optimized QA approaches to enhance the reliability of SBRT VMAT quality assurance reliability.

## Figures and Tables

**Table 1:** Slopes of linear regression lines between gamma passing rates (%) and MLC errors (mm). (% Slope values are expressed as % per mm).

	EPIQA	ArcCHECK SNCPATIENT	ArcCHECK 3DVH	COMPASS	Octavius 4D
1%/1 mm	−21.8	−17.10	−14.34	−17.83	−8.41
2%/1 mm	−17.98	−17.5	−11.49	−20.8	−9.16
1%/2 mm	−12.17	−14.97	−8.54	−14.99	−10.35
2%/2 mm	−9.86	−13.72	−5.82	−14.14	−9.6
3%/2 mm	−7.76	−11.26	−3.35	−12.97	−7.97
3%/3 mm	−3.9	−6.84	−1.78	−5.93	−6.55

**Table 2:** Pearson correlation coefficients and corresponding *p*-values between global and local gamma passing rates using the entire dataset.

Gamma Criteria	EPIQA		SNCPATIENT		3DVH		Octavius 4D	
	r	*p*	R	*p*	R	*p*	r	*p*
3%/3 mm	0.865	<0.001	0.283	0.171	0.573	0.003	0.759	<0.001
3%/2 mm	0.92	<0.001	0.314	0.126	0.390	0.054	0.78	<0.001
2%/2 mm	0.817	<0.001	0.580	0.002	0.419	0.037	0.814	<0.001
2%/1 mm	0.925	<0.001	0.368	0.071	0.247	0.234	0.674	<0.001
1%/2 mm	0.429	0.032	0.939	<0.001	0.689	<0.001	0.917	<0.001
1%/1 mm	0.621	0.001	0.865	<0.001	0.397	0.050	0.834	<0.001

**Table 3:** (**a**) AUC and *p*-value results for different detector systems according to different linac error types under 3%/3 mm gamma analysis. (**b**) AUC and *p*-value results for different detector systems according to different linac error types under 3%/2 mm gamma analysis. (**c**) AUC and *p*-value results for different detector systems according to different linac error types under 2%/2 mm gamma analysis. (**d**) AUC and *p*-value results for different detector systems according to different linac error types under 2%/1 mm gamma analysis. (**e**) AUC and *p*-value results for different detector systems according to different linac error types under 1%/2 mm gamma analysis. (**f**) AUC and *p*-value results for different detector systems according to different linac error types under 1%/1 mm gamma analysis.

**(a)**										
**3%/3 mm**										
	**EPİQA**		**SNCPATIENT**		**3DVH**		**COMPASS**		**Octavius 4D**	
	**AUC**	***p***	**AUC**	***p***	**AUC**	***p***	**AUC**	***p***	**AUC**	***p***
0.5° col	0.514	0.869	0.517	0.839	0.451	0.578	0.545	0.587	0.459	0.621
2° col	0.97	<0.001	0.740	0.004	0.636	0.126	0.518	0.823	0.502	0.985
0.25 mm	0.972	0.020	0.734	0.004	0.86	<0.001	0.462	0.641	0.698	0.016
0.5 mm	0.99	<0.001	0.939	<0.001	0.996	<0.001	0.494	0.938	0.88	<0.001
1 mm	1.000	<0.001	1.000	<0.001	1.000	<0.001	0.914	<0.001	0.997	<0.001
2 mm	1.000	<0.001	1.000	<0.001	-	-	1.000	<0.001	1.000	<0.001
**(b)**										
**3%/2 mm**										
	**EPİQA**		**SNCPATIENT**		**3DVH**		**COMPASS**		**Octavius 4D**	
	**AUC**	***p***	**AUC**	***p***	**AUC**	***p***	**AUC**	***p***	**AUC**	***p***
0.5° col	0.515	0.854	0.508	0.923	0.479	0.813	0.492	0.923	0.478	0.793
2° col	0.995	<0.001	0.690	0.021	0.646	0.100	0.499	0.992	0.475	0.764
0.25 mm	0.994	<0.001	0.745	0.003	0.861	<0.001	0.518	0.823	0.648	0.073
0.5 mm	0.994	<0.001	0.974	<0.001	1.000	<0.001	0.769	0.001	0.781	0.001
1 mm	1.000	<0.001	1.000	<0.001	1.000	<0.001	0.995	<0.001	0.987	<0.001
2 mm	1.000	<0.001	1.000	<0.001	-	-	1.000	<0.001	1.000	<0.001
**(c)**										
**2%/2 mm**										
	**EPİQA**		**SNCPATIENT**		**3DVH**		**COMPASS**		**Octavius 4D**	
	**AUC**	***p***	**AUC**	***p***	**AUC**	***p***	**AUC**	***p***	**AUC**	***p***
0.5° col	0.584	0.308	0.531	0.705	0.501	0.991	0.484	0.846	0.475	0.764
2° col	0.992	<0.001	0.660	0.052	0.653	0.086	0.494	0.946	0.486	0.869
0.25 mm	0.986	<0.001	0.741	0.003	0.878	<0.001	0.556	0.497	0.601	0.222
0.5 mm	0.987	<0.001	0.986	<0.001	1.000	<0.001	0.787	<0.001	0.751	0.002
1 mm	1.000	<0.001	1.000	<0.001	1.000	<0.001	1.000	<0.001	0.984	<0.001
2 mm	1.000	<0.001	1.000	<0.001	-	-	1.000	<0.001	1.000	<0.001
**(d)**										
**2%/1 mm**										
	**EPİQA**		**SNCPATIENT**		**3DVH**		**COMPASS**		**Octavius 4D**	
	**AUC**	***p***	**AUC**	***p***	**AUC**	***p***	**AUC**	***p***	**AUC**	***p***
0.5° col	0.612	0.174	0.547	0.567	0.467	0.713	0.450	0.541	0.488	0.884
2° col	1.000	<0.001	0.643	0.082	0.594	0.292	0.450	0.541	0.51	0.900
0.25 mm	1.000	<0.001	0.746	0.003	0.88	<0.001	0.770	0.001	0.586	0.299
0.5 mm	1.000	<0.001	0.958	<0.001	1.000	<0.001	0.962	<0.001	0.714	0.010
1 mm	1.000	<0.001	1.000	<0.001	1.000	<0.001	1.000	<0.001	0.949	<0.001
2 mm	1.000	<0.001	1.000	<0.001	-	-	1.000	<0.001	1.000	<0.001
**(e)**										
**1%/2 mm**										
	**EPİQA**		**SNCPATIENT**		**3DVH**		**COMPASS**		**Octavius 4D**	
	**AUC**	***p***	**AUC**	***p***	**AUC**	***p***	**AUC**	***p***	**AUC**	***p***
0.5° col	0.659	0.054	0.491	0.915	0.462	0.670	0.512	0.884	0.488	0.884
2° col	0.990	<0.001	0.611	0.177	0.575	0.400	0.523	0.778	0.479	0.801
0.25 mm	0.976	<0.001	0.719	0.008	0.886	<0.001	0.604	0.207	0.589	0.282
0.5 mm	0.965	<0.001	0.976	<0.001	1.000	<0.001	0.826	<0.001	0.745	0.003
1 mm	1.000	<0.001	1.000	<0.001	1.000	<0.001	1.000	<0.001	0.978	<0.001
2 mm	1.000	<0.001	1.000	<0.001	-	-	1.000	<0.001	1.000	<0.001
**(f)**										
**1%/1 mm**										
	**EPİQA**		**SNCPATIENT**		**3DVH**		**COMPASS**		**Octavius 4D**	
	**AUC**	***p***	**AUC**	***p***	**AUC**	***p***	**AUC**	***p***	**AUC**	***p***
0.5° col	0.738	0.004	0.520	0.808	0.484	0.859	0.451	0.554	0.505	0.954
2° col	1.000	<0.001	0.641	0.088	0.613	0.205	0.450	0.548	0.500	1.000
0.25 mm	1.000	<0.001	0.744	0.003	0.914	<0.001	0.762	0.002	0.581	0.327
0.5 mm	0.976	<0.001	0.984	<0.001	1.000	<0.001	0.959	<0.001	0.716	0.009
1 mm	1.000	<0.001	1.000	<0.001	1.000	<0.001	1.000	<0.001	0.949	<0.001
2 mm	1.000	<0.001	1.000	<0.001	-	-	1.000	<0.001	1.000	<0.001

## Data Availability

The data presented in this study are available from the corresponding author upon reasonable request. The data are not publicly available due to privacy and ethical restrictions.
